# Benefits of Outdoor Sports for Society. A Systematic Literature Review and Reflections on Evidence

**DOI:** 10.3390/ijerph16060937

**Published:** 2019-03-15

**Authors:** Barbara Eigenschenk, Andreas Thomann, Mike McClure, Larissa Davies, Maxine Gregory, Ulrich Dettweiler, Eduard Inglés

**Affiliations:** 1 TUM Department of Sport and Health Sciences, Technical University Munich, Georg-Brauchle-Ring 60/62, 80992 München, Germany; andreas.thomann@tum.de; 2 Sport Northern Ireland, c/o Tollymore National Outdoor Centre, 32 Hilltown Road, Bryansford, Newcastle BT33 0PZ, UK; mikemcclure@sportni.net; 3 Sport Industry Research Centre, Sheffield Hallam University, Sheffield S10 2BP, UK; l.e.davies@shu.ac.uk (L.D.); m.gregory@shu.ac.uk (M.G.); 4 Universitetet i Stavanger, Kjell Arholms gate 41, 4021 Stavanger, Norway; ulrich.dettweiler@uis.no; 5 National Institute of Physical Education of Catalonia (INEFC), University of Barcelona (UB), Av. Estadi 12-22, 08038 Barcelona, Spain; eduard.ingles@gencat.cat

**Keywords:** outdoor sports, outdoor recreation, health enhancing physical activity, social benefits and costs or social impacts, outdoor education

## Abstract

The combination of physical activity and being in nature is recognized as providing a range of significant benefits. The objective of this literature review was to compile an overview of the social benefits and costs associated with outdoor sports within the academic literature and to reflect on the quality of underlying evidence that supports the relationship. A systematic review was carried out with seven partners from different European countries, including Bulgaria, France, Germany, United Kingdom, Italy, Portugal, and Spain. From a total of 17,560 studies identified, 133 studies were selected with relevant data extracted to standardized forms. The selected studies have been analyzed with qualitative research methods. A meta-analysis could not be conducted due to the heterogeneity of the study designs and outcome measures. As a result, the review gives an overview of the social impacts associated with outdoor sports which have been clustered to six broad categories: physical health, mental health and wellbeing, education and lifelong learning, active citizenship, crime reduction, and anti-social behavior, as well as additional benefits. The review furthermore revealed gaps in the evidence base which are especially notable in the long-term effects that outdoor sports can have on personal and social development.

## 1. Introduction

There is widespread knowledge and a body of evidence-based research on the importance of physical activity especially for physical and mental health and wellbeing [1]. Furthermore, there is growing evidence on the benefits for people of being in nature or having contact with the natural environment [2]. Consequentially, physical activity that is carried out in nature is discussed for bringing together those positive impacts and even having synergistic effects. In this context, experts from different fields have highlighted the benefits of outdoor sports that often go beyond being active in a non-natural environment [3] (The term sport is used interchangeably with physical activity and based upon the inclusive, broad definition of the Council of Europe (1992) that describes sport as “all forms of physical activity which, through casual or organised participation, aim at expressing or improving physical fitness and mental well-being, forming social relationships or obtaining results in competition at all levels” [4]).

Beyond the health enhancing effects of physical activity and nature, outdoor sports are also associated with social benefits including the intra- and interpersonal development for young people, crime reduction, and active citizenship as they provide unique opportunities within the natural and social environments. They connect individuals with nature, with other people and with themselves [5] and so achieve a range of positive effects simultaneously.

In the context of urbanization, insufficient activity levels, sedentary behavior, and an increasing disengagement between people and the natural environment, it raises the question if and how outdoor sports can be part of the solution. However, there is a gap in the evidence base to better understand the benefits of outdoor sports as a whole and therefore support investment in health enhancing physical activity (HEPA) in the natural environment.

This systematic literature review raises two questions: what kind of social benefits are associated with outdoor sports within the academic literature and what quality of underlying evidence exists that supports the association.

## 2. Methods

To identify the social impacts of outdoor sports a systematic literature review was carried out with seven partners from different European countries involved, including Bulgaria, France, Germany, Great Britain, Italy, Portugal, and Spain.

Previous literature has analyzed the social impacts of sport in general like Coalters review on the social benefits of sport [6] or the Culture and Sport Evidence programme which identified outcomes related to health, subjective wellbeing, crime, education, and social capital [7]. Other reviews also dealt with the benefits of outdoor adventure activities [5] or highlighted the specialties of physical activity in a natural environment [3] and therefore served as a kind of core text.

From previous research a list of outcomes relating to outdoor sport were identified. Those impacts included but were not restricted to the following: (1) Physical and mental health and well-being; (2) Education and life-long learning; (3) Active Citizenship including social inclusion, integration, volunteering and community cohesion; (4) Reduction of crime and anti-social behavior.

### 2.1. Literature Identification

A systematic literature review was carried out using the following databases: SportDiscus, ERIC, benefitshub, PubMed, SURF, Cobiss, Natursportinfo, B-On, Dialnet, Share, Data bases of INSEP (Institut National du Sport, de l’Expertise et de la Performance), ENVSN (Centre de documentation de l’Ecole Nationale de Voile et des Sports Nautiques), ENSA (École Nationale de Ski et d’Alpinisme), PRNSN (Pôle Ressources National des Sports de Nature), Catalogue SUDOC and the catalogue of National Sports Academy Sofia. The search was based on title and abstract and used a special keyword combination set as the search formula. The timeframe was limited to 15 years from March 2002 until March 2017. If possible, filters were set for academic journal articles only.

The search string formula (see Table 1) consisted of three parts: (1) an element of nature or outdoor combined with (2) forms of active, physical exercise and (3) the description of effects. Those effects were described as a keyword combination of benefits, impacts, or costs in general and the anticipated benefits that were identified through former literature reviews. To avoid a possible bias the search string consisted not only of terms like “benefit” but also included neutral words like “impact” or “effect” or negative phrases like “costs”.

A second search string was created that used a keyword combination of single outdoor sport activities and combined this with the possible outcomes. The two search strings are listed in Table 2.

The search strings were translated in all languages of the partner countries using the same key words or equivalents respecting national practice. As equivalents of outdoor sport, terms like “deporte en la naturaleza” or “en el medio natural” were for example included within the Spanish search and different naming of disciplines like horse riding or horseback riding was taken into account. Inclusion criteria were defined according to the underlying definition of outdoor sports that had been agreed within the partners for the use of the project.

The search included activities:

(1) that are normally carried out with a (strong) relation to nature and landscape and the core aim is dealing with natural elements rather than with an object
(2) where the natural setting is perceived by users, as at most, only minimally modified by human beings
(3) that are perceived as physically demanding
(4) that are based on man or natural element power and are not motorized during the sport itself
(5) that may use some form of tool (for example a surf board, bicycle, skis etc.) or just involve the human body
(6) It may include activities that have their roots in natural places but use artificial structures designed to replicate the natural environment.

Examples of activities that are incorporated under this definition are hiking, trekking, swimming in the natural environment, cycling, snowshoeing, cross-country skiing, canoeing, surfing, or climbing. Sports and activities that take place outdoors or in open air but do not take place in a natural environment like football or tennis are not included in the definition. Also excluded are activities that are not physically demanding like camping or bird watching as well as motorized activities like jet skiing or activities that focus on an object like, for example, a kite or a ball. A list of examples of included and excluded activities is available as an online supplement (see Table S1).

Additionally, the following inclusion criteria were defined for the selection process: (1) interventions with significant nature and sport experience according to the definition of outdoor sport, (2) academic relevance (methodological quality criteria), (3) relationship between outdoor sport intervention and social benefit. Examples for exclusion are articles on management advice, guidelines and examples for programs/training, studies on outdoor play or on open space activities like gardening.

An additional search in reference lists of selected articles was not conducted. Besides the systematic search in the data bases which focused on journal articles, additional studies e.g. of other publication types were included if they were rated as very relevant for the topic. This included grey literature including reports or congress contributions which are not part of the traditional commercial or academic publishing and distribution channels. However, as they were not part of the systematic process they were collected and analyzed separately.

### 2.2. Screening and Data Extraction

Taking all databases and partner searches together, a total number of 20,950 records were identified in the primary search. After deletion of duplicates the sum of primary data reduced to 17,560 studies that were then screened for inclusion and exclusion. As a first step, the studies were screened by title and abstract. The researchers consulted the full text as a second step for definite selection. Per country, at least two researchers reviewed and discussed the selection. After the national selection process, the literature review leaders additionally controlled the studies centrally for inclusion and quality criteria.

**Figure 1:**
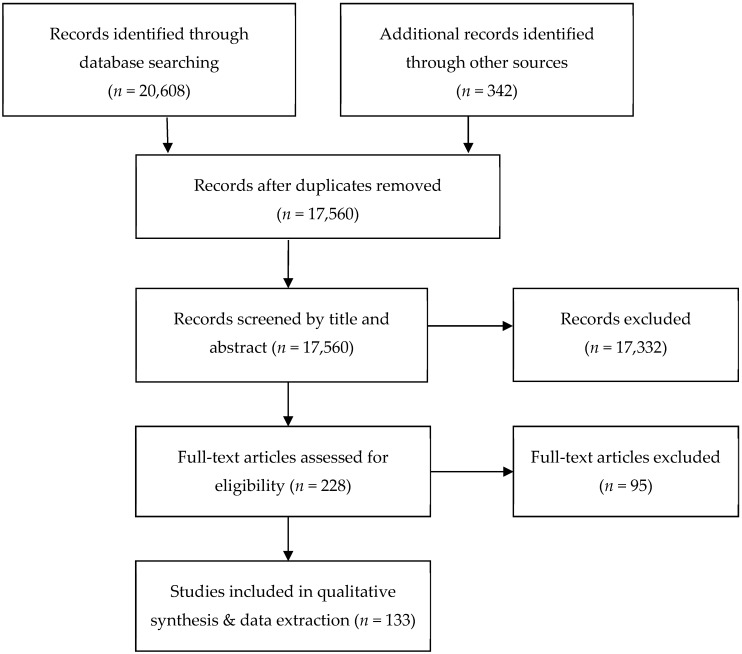
Research and selection process.

Following this process (see Figure 1), 133 studies were selected and standardized forms were developed to extract relevant data from these selected studies. This data extraction was made by full texts and included the following data: full reference, title in English, methodological design, country, sample description (age, number of participants, population), description of study and interventions, type of sport, key findings, description of social benefits and outcomes, quantification of results and methods used. A table with relevant extracted data of all selected studies is available as an online supplement (see Table S2).

## 3. Results

Overall, 133 studies were selected and analyzed. The majority of the selected studies (61.7%) originated in English speaking countries, with over a quarter of studies from the U.S.A. (26.3%), 7.5% from Canada, and 8.3% from New Zealand and Australia. Overall, 72 studies (54%) came from European countries, with 46 non-English studies and 26 from Ireland and Great Britain. A table on the origin of selected studies is available as an online supplement (see Table S3).

### 3.1. Description of Benefits

The benefits were grouped into six broad categories including physical health, mental health and wellbeing, education and life-long learning, active citizenship, crime-reduction, and anti-social behavior as well as additional benefits.

Table 3 summarizes the research design of the studies reviewed, grouped by each benefit identified. The first number is the total number of studies reviewed and the number in brackets shows the number of studies identified through the non-systematic approach (additional studies in scientific journals, grey literature). For example, there were six longitudinal studies related to education and life-long learning. Of these, three studies were additional studies found in scientific journals and one study was from the grey literature From the table it can be seen that topics such as health and education are more prevalent and evidence-based literature is rare for other types of benefits. The majority of studies (74 articles) deal with the effect of outdoor sports on mental health benefits followed by the effects on education and life-long learning (57 studies) and physical health (46 studies).

#### 3.1.1. Physical Health Benefits

As per physical activity in general, outdoor sports are associated with a range of positive health benefits. This includes general health related factors [5,8,9,10,11,12,13,14,15,16,17,18,19,20,21,22,23,24,25,26,27,28,29,30,31,32,33] such as increased fitness and better cardiovascular function, as well as reduced blood pressure, obesity, resting heart rate, and a positive influence on other health markers. Those health-enhancing effects result in a reduced risk for several major diseases [12,28,32,34,35,36,37] like heart attack [35], 13 types of cancer [34], stroke, and type 2 diabetes [32]. Leisure-time physical activity however is also associated with higher risks of malignant melanoma and prostate cancer [34]. While prostate cancer showed only a slightly increased hazard ratio of 1.05 (95% CI, 1.03–1.08) and the authors state that the effect may furthermore be influenced by prostate screening bias, the hazard ratio of malignant melanoma is 1.27 (95% CI, 1.16–1.40). This risk is seen as an effect of outdoor activity, which is often practiced in light clothing and therefore was associated with a substantially increased risk of sunburn.

While outdoor sports are often viewed as synonymous with a higher risk of injuries and cases of death, this could not be confirmed through this research [31,32,38,39,40,41,42,43,44]. It has to be noted that there are certain types of injuries that are more prevalent for specific sports compared to the general trauma population such as higher rates of spino-pelvic injuries in paragliders [43]. However, inactivity is identified as the reason for premature deaths and a shorter life-expectancy [32].

Besides the reduction of diseases, outdoor sports are associated with a better subjective overall health perception [45,46] and a better physical quality of life. In the context of healthy ageing [28,32,33,47,48,49], it was shown that outdoor sports can help the elderly to maintain their physical performance [48]. Furthermore, the exposure to sun helps to maintain the level of vitamin D (25 OHD level) especially in the elderly [50]. Outdoor activities are also discussed as helping to prevent multiple sclerosis [51] and the onset and progression of myopia [52].

Regarding the data quality and the methodological design (see Table 4), general health related factors are often based on cross-sectional data and longitudinal studies, however the research design of a meta-analysis and randomized controlled trials (RCT) were also applied. Within the research on diseases, pre-post measurements are the predominant methods.

#### 3.1.2. Mental Health and Wellbeing Benefits

The majority of published articles (74 studies) that were identified through this review, focused on the benefits that outdoor sports can create for mental health. This shows a high research interest in dealing with mental disorders and also the various opportunities that outdoor sports can provide to help to prevent and cure mental health problems (see Table 5). Several research teams highlight the positive effects on general mental health and psychological stability of being active in the natural environment [5,11,17,18,20,25,31,33,53,54,55,56,57,58]. Evidence was also provided of impacts on overall wellbeing, quality of life, happiness, and life satisfaction [5,14,15,20,31,33,45,46,48,53,58,59,60,61,62,63,64,65,66]. Overall, green and blue environments seem to have especially positive effects that go beyond the benefits of being physically active in a non-natural environment. In this context, Thompson, Coon, Boddy, Stein, Whear, Barton, and Depledge [3] conducted a systematic review of the comparative effects of participating in indoor and outdoor activity that confirms these effects. Reported effects of exercising in natural environments were that participants had greater feelings of revitalization and positive engagement, decreases in tension, confusion, anger, and depression.

Studies that analyze the influence of outdoor sport and recreation on special affective states show positive effects for mood, resilience, feelings of revitalization, and positive engagement as well as restoration for people living in cities [3,9,10,14,22,33,61,65,67,68,69,70,71,72,73,74,75,76,77]. Negative affective states like stress, depression, anxiety, tension, confusion, anger, rumination, loneliness, and neuroticism could be reduced by participation in outdoor sports [3,10,18,24,33,46,53,59,61,63,65,68,73,74,76,77,78,79,80,81,82,83,84]. Furthermore, many positive experiences are described [22,62,67,68,75,79,85,86,87] such as pleasure and enjoyment, meditation, independence, basic psychological needs of autonomy, competence and relatedness, experiences of flow, comfort and intense emotions, enhanced feeling of body, discovering the pleasure of achievement, vital strength and a higher will to live in drug addicts, and of course an intense nature experience. As a possible negative effect, feelings of calmness and tranquility may be decreased following outdoor exercise [3].

Overall, there were 32 studies on the impact of outdoor sports and recreation on affective states, 19 on positive and 23 on negative ones. This shows a high level of research activity and interest in this field. Also, the number of higher quality research with meta-analysis, RCTs, and case-control studies is significant (see Table 5). Studies that evaluated more general effects like the overall wellbeing and quality of life as well as positive experiences do not provide this quality of data.

The review identified and analyzed 27 studies that dealt with the effect of outdoor activities and sports on self-development in relation to mental health benefits [5,9,22,59,60,63,64,67,68,69,70,71,72,88,89,90,91,92,93,94,95,96,97,98,99,100,101]. This includes effects of increased self-esteem, self-efficacy, social effectiveness, self-confidence, and a better self-concept. The typical research design is a case-control study on the one hand but also a large number of qualitative insights from the field that support the positive effects of outdoor sport on self-development on the other.

Outdoor sports are not only related to influence the affective states, practitioners also seem to develop better control of affective states and coping strategies [5,31,46,81,84,91,92,98]. Eight studies within the selected sample dealt with personal control, increased sensitivity to one’s own well-being, self-regulation, effects of emotional coping, or a better stress management. Better coping strategies and improved self-regulation have also been described in specific groups such as teenagers with attention deficit hyperactivity disorder (ADHD) [91] or veterans [92].

Besides the more functional perspective of ageing, outdoor sports also provide a rich resource for active and happy ageing [32,47,76,102] with effects like positive engagement, revitalization, tranquility, and increased mood in the elderly. It is clear that sport involvement can even have an emotional component as described in Minello and Nixon’s [47] study on older men participating in cycling with the title “‘Hope I never stop’: older men and their two-wheeled love affairs”.

Outdoor sports are not only presented as supporting the prevention of mental illnesses [32,33], they are also used in the treatment of them, with prominent examples like Alzheimer’s disease [32], dementia [33], or major depressive disorders [74]. Furthermore, physical activities and sports in the natural environment are also used as a therapeutic tool for a range of groups with specific needs such as children with disabilities [93], persons with disabilities after acute injuries [63], veterans [92], MS patients [103], ADHD sufferers [88,104,105], young people at risk and disorderly adolescents [106], dementia patients [33], or drug addicts [85].

#### 3.1.3. Benefits in Education and Life-Long Learning

Outdoor sports provide an environment that leads to an intense contact with oneself, others and nature and therefore are discussed for having impacts on interpersonal and intrapersonal development as well as influencing the relation of humans with nature (see Table 6). Overall, 57 studies have been identified in the category of education and life-long learning.

Intrapersonal development was highlighted in the literature as being about the physical, mental, cognitive, emotional, social, behavioral, and spiritual aspects of self [5,14,17,18,22,25,31,59,60,64,69,84,85,88,89,90,91,93,95,97,100,101,106,107,108,109,110,111,112,113,114,115,116]. It includes personal skills and improved motor skills, an increased emotional intelligence, personal responsibility, mindfulness and an enhanced spiritual, sensory, and aesthetic awareness. The intense contact with one’s self in nature also leads to a better self-knowledge and understanding of oneself, and has a positive impact on self-esteem, self-efficacy, and self-actualization. Furthermore, outdoor sports are associated with increased self-motivation and show positive effects on volitional qualities, assertion and inner strength, endeavor and readiness to face challenges. These qualities can also have effects that go beyond the sport activity and influence educational achievements. Six studies showed an association between outdoor sport programs and educational performance and motivation [5,18,60,107,112,116]. This included an increase in sense of purpose for learning and motivation to study, a higher engagement within lessons, better academic learning, efficacy, and better achievements.

Furthermore, being active in the natural environment does not only influence the attitude towards learning, it also has effects on cognitive aspects [17,32,56,74,80,88,104,117,118] such as attention [56,118] and memory span [74,80], brain structure, function, and connectivity [117], as well as intellectual flexibility and problem-solving-skills. Those cognitive aspects are also important in the context of healthy ageing and preventing cognitive decline [32,117].

While the described cognitive effects rely mainly on RCTs and case control studies, it needs to be noted that the effect on educational motivation and achievements was mainly based on qualitative data from the field and literature reviews. Benefits for personal development are based on a large number of case studies and expert evaluations from the field (15 qualitative studies) but are also backed up by case-control studies (8) and longitudinal research designs (4).

As outdoor sports often involve groups or settings where it is necessary to work together, they also lead to various interpersonal or group benefits [5,13,14,18,25,31,32,60,64,85,88,106,107,109,111,115,116,118,119,120,121,122]. Interpersonal development was characterized by increased communication skills, cooperation and social interaction, enhanced relationships, responsibility, empathy, engagement, social trust, and better overall group cohesion.

Connecting people with nature and the resultant improved understanding of the relationship to the environment and our dependency on it are further key facets of outdoor sports. Overall 18 studies highlighted positive effects on environmental awareness, attitudes, and behavior [5,18,20,25,31,95,99,100,101,115,123,124,125,126,127,128,129,130], however a direct causality of better environmental behavior is rarely evidenced [123]. Described environmental aspects included an increased connectedness to nature, awareness, sensitivity, and empathy as well as positive effects on environmentally responsible behavior and stewardship. Building upon this positive relationship, outdoor sports are seen as an important tool for environmental education by many experts and scientists. As nature-based sport can go beyond the mere transmission of knowledge it has the potential to enhance pro-environmental behavior in the context of situated and experiential learning [128]. Some authors described how outdoor sports could be used as an attractive and motivational method to interest young people in the topic of sustainability and to teach and understand this complex construct in an appealing way with a lasting effect [129]. However, it has to be mentioned, that only seven of the 18 studies are built upon quantitative data and there is still a lack of research that adequately evaluates the long-term effects stated by the practitioners.

Besides the positive effect of connecting people with nature, there are some potential negative impacts of damage to the environment or disturbance of wildlife in sensitive natural areas [20], due to the locations for many sport activities.

#### 3.1.4. Active Citizenship

As outdoor sports provide opportunities and places for social interaction, contacts, and relations they can lead to increased social connectedness and are therefore associated with various benefits of active citizenship (see Table 7). This includes volunteering [29] and community benefits [5,64,101,110,122,131,132,133,134] such as the construction and maintenance of local community life, identity, and pride [110,134]. In this context, outdoor sports are also described as a contributor to bonding capital for families, groups, and communities [5,53,79,97,121].

Outdoor sports programs also showed positive effects in the inclusion or (re-)integration of special groups including individuals with physical and/or mental disabilities [13,15,26,63,64,108,135], young people with autism [120], disengaged youths [107], or for the reintegration of youth at risk [106]. Also elderly, who are often at risk of social isolation, can benefit from participation in outdoor activities [53].

Overall, the benefits within active citizenship are less clearly evidenced than the categories described previously. Only five longitudinal studies were found to provide quantitative data.

#### 3.1.5. Crime Reduction and (Anti-)Social Behavior

Only 11 studies focused on the benefits for crime reduction [5,20,31,85] and prosocial behavior [60,88,95,106,119,121,131] and the evidence-base is primarily qualitative and anecdotal reports from the field (see Table 8). However, the studies found give valuable insights as to how outdoor sports can be used to increase prosocial behavior [119], reduce smoking, alcohol and substance misuse [31], or prevent youth delinquency [20]. Experiences of controlled risk are furthermore discussed as a mechanism to help to improve the behavior and habits of adults with drug addiction or other social exclusion factors [85].

In this context, outdoor sport programs were implemented for children and young people in foster care institutions [106] as well as for disaffected youths and pupils showing anti-social behavioral traits [60] with the result of a decrease in the number of behavioral referrals.

#### 3.1.6. Additional Benefits

Two aspects have been described as additional results in many studies. However, those effects are not only an add-on, they also build the basis of a successful implementation of the benefits described above.

One important aspect that has been underlined in many studies and project evaluations (see Table 9) was that outdoor sports support physical activity throughout the entire life course [3,11,14,23,27,28,31,41,47,58,68,75,79,91,95,101,106,118,136]. Outdoor sports were shown to be used as a tool to successfully activate sedentary, non-active people, promote active and healthy lifestyles, and are able to influence positive attitudes towards physical activity. As outdoor sports have connections to lifetime activity-habits, they can foster sport adherence over the life course and help people to find and maintain an active way of life. In a systematic review, Thompson, Coon, Boddy, Stein, Whear, Barton, and Depledge [3] raised the question as to whether physical activities in outdoor settings are more beneficial compared to indoor ones. The authors found that participants of outdoor sports reported greater enjoyment and satisfaction with the activity. They also declared a greater intention to repeat the activity at a later date.

Another important aspect is the accessibility [11,32,73,132] of most outdoor sports as they are predominantly free at the point of use and have few limitations to participation. In the context of urban recreation, outdoor sport venues like parks, forests, and other green spaces, as well as local rivers or lakes are easy to access and can be seen as low-cost opportunities that are open for all and are valued by people with a low-income [11].

Other effects [20,29,30,32,115,131,137,138] described economic impacts, contributions to rural economy, cost-effectiveness, or philosophical statements such as sport for development and peace or “worldmaking”.

## 4. Discussion

The aim of this review was to show the broad overview of impacts associated with physical activity in nature. Based on a qualitative clustering, effects were grouped into the six categories of physical health, mental health and wellbeing, education and life-long learning, active citizenship, crime-reduction, and anti-social behavior, as well as additional benefits. The broad analysis of studies also showed the various contexts in which outdoor sports are practiced and successfully implemented. It highlighted that they can bring benefits for young as well as old people, people from different financial backgrounds or with special needs, those living in urban areas as well as those from rural areas. However, differences in the impact of outdoor sport for different population subgroups were not addressed within this article and the validity of the described benefits were not proven for all ages, subgroups, or different sport conditions. Furthermore, frame conditions like duration, intensity, or sun exposure, which can vary a lot for different countries but also within individual dispositions, need to be taken into account.

### 4.1. Methodological Limitations and Risk of Bias

Due to the broad research scale and the heterogeneity of the research team, the study design had to deal with some methodological limitations and cannot provide the detail of a more focused analysis. The review included international as well as national databases and translated the search string in seven different languages. Therefore, the central quality controlling could only be conducted for the inclusion of studies. The exclusion criteria could not be controlled by the project leaders. As not all the partners used the literature program endnote due to incompatible or even analogue databases and varying working practice, reasons for exclusion were not documented for all studies and therefore are not reported within the overall review.

Through the creation of a search string that included neutral as well as negative key words, there was an attempt to avoid bias. However, it has to be acknowledged that the research team consisted of persons with an outdoor sport related biography and the qualitative studies relying on experts from the field are subject to a higher risk of bias. The assessment of risk of bias was not analyzed for every single study.

### 4.2. Limitations in Evidence Base

Due to the heterogeneity of studies and outcomes, no summary measures were made. Regarding the data quality, it can be concluded that evidence is not equally strong for every type of benefit. Additionally, frame conditions like ethical limitations or special populations, as well as the complexity of research questions, vary a lot. Overall, there is stronger evidence for positive effects of outdoor sports on physical and mental health especially for affective states and self-development. Within the category of education and lifelong learning, benefits for personal and social development are primarily based on case-control studies and longitudinal research designs as well as on a large number of qualitative insights from the field. Benefits on active citizenship are less clearly evidenced and the review also shows a paucity of good quality studies on crime reduction and prosocial behavior.

Overall, only limited negative effects were found within this literature review. Those included a higher risk for malignant melanoma and prostate cancer [34], feelings of reduced calmness following outdoor exercise [3] as well as injuries that are more prevalent for special sport disciplines, like a higher rate of serious spinal and pelvic injuries due to airborne sports [43] or specific mountain biking injuries [44]. Despite the described positive effects on the relationship to nature, it has to be mentioned, that there is also the potential for activities to have a negative impact on the natural environment. With high numbers of people moving in vulnerable areas, visitor concepts and education are needed to avoid damage to the natural environment. Potential social conflicts between different nature sports activities also need to be taken into account [137].

The example of skin cancer and potential environmental damages are examples that highlight that both positive and negative impacts may occur simultaneously. Benefits like a higher level of vitamin D are for example in opposition to a higher risk of malignant melanoma. The studies found often focus on special correlations like the effect of sun exposure on vitamin D status or multiple sclerosis but do not take into account the possible negative effects sufficiently. Overall, the negative impacts need further research.

### 4.3. Limitations Regarding Data Sources

In the field of outdoor sport, our research highlighted that a lot of project reports or high-quality interventions are not published within the academic journals but only in grey literature media. As these studies were not part of the systematic process they were collected and analyzed separately.

The question for publication media and data bases also needs to be reflected critically regarding the negative impacts that were found within this research. As those were very limited, other data bases and ways of research may be more adequate for addressing specific impacts like environmental damage. Those were only mentioned as a hypothetic impact within the studies found or from the subjective perception of practitioners and residents [137]. However the public discussion of e.g. over-mountaineering and over-use in general are very present. Within the effects of injuries, low risks have been reported for some sports but also the description of special types of injuries including those attributed to airborne sports. An examination of accident statistics of the relevant national organizations is likely to be more insightful than the research conducted within this review.

### 4.4. Implications for Stakeholders

Although this review revealed limitations in the evidence base and the interpretation and extrapolation of the findings was restricted by the heterogeneity of outcome measures and the methodological quality of the available evidence in some effect categories, the findings are positive. Without giving recommendations, implications can be made for various stakeholders. From a practitioner’s point of view, outdoor sports provide significant physical and mental health benefits that often go beyond the benefits of physical activity indoors. Physical activity in a natural environment seems to be an effective answer to actual problems like stress, depression, and a range of diseases or conditions associated with modern life. In a school context, outdoor sport programs are described as very valuable for the development of young people, personally but also in a group context. Therefore, outdoor sports are utilized in various programs including sustainable development education or the (re)integration of special groups such as those with disabilities. They help to connect people with the communities they live in and build up strong connections to other people, places, and nature. From a decision maker’s point of view, investments in outdoor sports are estimated as being very cost-effective, as many positive effects are reached simultaneously and no significant infrastructure is required—as the natural environment provides the arena for this.

## 5. Conclusions

This systematic review provides an overview on the social benefits associated with physical activity in nature. Rather than analyzing certain treatments and outcome measures, the aim was to show the effects from a qualitative point of view and present the complexity and multiple layers of benefits. A qualitative clustering was conducted that grouped the effects into the six categories of physical health, mental health and wellbeing, education and life-long learning, active citizenship, crime-reduction, and anti-social behavior as well as additional benefits.

The evidence reveals that outdoor sports are linked to achieving multiple outcomes und they help people to find and maintain a lifetime physical activity. As outdoor sports are accessible and appealing to a very broad audience, the benefits gained are also open for all kinds of stakeholders. Furthermore, as many benefits can be reached simultaneously, outdoor sports do not only benefit the practitioners in multiple ways but from a decision maker’s point of view, investments in outdoor sports are seen as being very cost-effective.

Effects are presented for a broad range of physical and mental health outcomes but also for the personal and social development for individuals and groups and for benefits affecting communities as a whole. However, the evidence base is not equally strong for each type of benefits. While there are some higher-quality studies in relation to health issues, the evidence on benefits relating to psychological and social effects are less clearly evidenced except for some aspects like self-development. Effects on active citizenship and crime reduction do lack a strong evidence base and are primarily supported by reports and qualitative evaluations from the field.

More research is needed with large, well designed studies and also to analyze the long-term effects and sustainability of programs. Furthermore, research is especially needed in the field of social effects like education, active citizenship, and (anti-)social behavior.

## Figures and Tables

**Table 1:** Key words for creation of search string formula.

Nature Relation		Physical Activity		Outcome
Nature or outdoor	AND	Sport or activity or exercise	AND	—benefit, impact, effect, cost or —health, wellbeing or —education, lifelong learning, personal and social development, environmental awareness, social capital, concentration and thinking skills, cognitive development or —inclusion, integration, gender equality, volunteering, community involvement, cohesion, social, bridging and bonding capital, connectedness or —prevention/ reduction of crime, pro social and anti-social behavior, youth justice, criminal incidents, vandalism

**Table 2:** Search string formulas used.

**Search String 1**
(outdoor * OR nature *) AND (sport * OR active * OR exercise *) AND (benefit * OR impact * OR effect * OR cost * OR health OR well-being OR wellbeing OR educat * OR learn * OR knowledge OR environment * OR develop * OR self * OR social OR personal OR cognitive OR citizenship OR inclusi * OR integrat * OR volunt * OR gender OR disab * OR migrant * OR relationship * OR network OR cohesion OR community OR capital OR crim * OR vandal * OR justice)
**Search String 2**
(water? sport OR swimming OR rowing OR sailing OR kayaking OR canoeing OR surfing OR coasteering OR rafting OR diving OR canyoning OR snow? sport OR snowboarding OR skiing OR ski? touring OR snow? shoeing OR hiking OR mountaineering OR mountain? sports OR trekking OR climbing OR paragliding OR horse? riding OR cycling OR * biking) AND (benefit * OR impact * OR effect * OR cost * OR health OR well-being OR wellbeing OR educat * OR learn * OR knowledge OR environment* OR develop* OR self * OR social OR personal OR cognitive OR citizenship OR inclusi * OR integrat * OR volunt * OR gender OR disab * OR migrant * OR relationship * OR network OR cohesion OR community OR capital OR crim * OR vandal * OR justice)

**Table 3:** Design of evidence per benefit ^1^.

Methodological Design	Physical Health (*n* = 46)	Mental Health & Well-Being (*n* = 74)	Education & Lifelong Learning (*n* = 57)	Active Citizenship (*n* = 23)	Crime Reduction (*n* = 11)	Additional Benefits (*n* = 46)
Systematic reviews & Meta-analyses	4 (0/1)	6 (0/2)	1 (0/1)	1 (0/1)	1 (0/1)	1 (0/0)
Randomized Controlled Trials	5 (0/0)	10 (0/0)	4 (1/0)	-	-	2 (0/0)
Case-control studies	3 (0/0)	14 (0/2)	14 (0/2)	-	2 (0/0)	9 (0/0)
Longitudinal studies	3 (0/0)	9 (2/2)	6 (3/1)	2 (2/0)	1 (1/0)	3 (1/1)
Cross-sectional surveys	11 (0/0)	9 (1/1)	2 (0/0)	1 (0/0)	-	4 (0/1)
Case reports & qual. evaluations	9 (0/1)	18 (0/2)	20 (0/2)	16 (0/2)	4 (0/0)	16 (0/1)
Economic evaluations	2 (0/2)	-	-	1 (0/1)	-	2 (0/2)
Non-systematic literature review	9 (0/3)	8 (0/2)	7 (0/2)	1 (0/0)	3 (0/1)	6 (0/3)
Policy statement, theoretical paper	-	-	3 (0/1)	1 (0/0)	-	3 (0/1)

^1^ The number in brackets shows the number of studies that did come up through the non-systematic approach (additional studies in scientific journals/ grey literature) within the total number.

**Table 4:** Design of studies within physical health impacts ^1^.

Design	General Health Related Factors	Diseases	Injuries & Life Expectation	Healthy Ageing	Subjective Health Perception	Sun Exposure Benefits
Overall	27	7	9	6	2	3
Meta	1	0	1	0	0	0
RCT	3	1	0	1	0	0
Case Control	1	0	0	0	0	1
Longitudinal	6	5	2	2	0	1
Cross-sectional	6	1	2	0	2	1
Qualitative data ^2^	5	0	2	3	0	0
Literature reviews ^3^	5	0	2	0	0	0

^1^ Studies may refer to one or more impacts, therefore the total sum can be higher than the number of studies within the overall impact category; ^2^ Qualitative studies include evaluations of case studies or interventions with qualitative research methods and expert opinion based on field experience; ^3^ Literature reviews are not based upon empirical data. RCT = randomized controlled trials.

**Table 5:** Design of studies within mental health and wellbeing impacts ^1^.

Design	General Mental Health Status	Quality of Life & Overall Well-Being	Mental Illnesses & Diseases	Positive Affective States	Negative Affective States	Control & Coping	Self-Development	Pos. Experiences	Active & Happy Ageing
Overall	14	19	2	19	23	7	27	9	4
Meta	0	0	0	3	4	0	1	0	0
RCT	2	0	0	3	4	0	2	0	0
Case Control	1	2	0	5	5	2	9	2	1
Longitudinal	2	6	1	5	4	1	4	0	1
Cross-sect.	3	5	0	0	2	1	0	1	0
Qual. Data ^2^	3	4	1	2	3	3	8	4	2
Lit. reviews ^3^	3	2	0	1	1	1	3	2	0

^1^ Studies may refer to one or more impacts, therefore the total sum can be higher than the number of studies within the overall impact category; ^2^ Qualitative studies include evaluations of case studies or interventions with qualitative research methods and expert opinion based on field experience; ^3^ Literature reviews are not based upon empirical data.

**Table 6:** Design of studies within educational impacts ^1^.

Design	Personal Development	Inter Personal Development	Educational Motivation & Achievements	Cognitive Aspects to Improve Learning	Environmental Aspects
Overall	33	22	6	9	18
Meta	0	0	0	0	0
RCT	0	1	0	4	0
Case Control	8	2	1	2	2
Longitudinal	4	4	0	1	4
Cross-sectional	1	0	0	1	1
Qualitative data ^2^	15	11	3	0	6
Literature reviews ^3^	5	4	2	1	5

^1^ Studies may refer to one or more impacts, therefore the total sum can be higher than the number of studies within the overall impact category; ^2^ Qualitative studies include evaluations of case studies or interventions with qualitative research methods and expert opinion based on field experience; ^3^ Literature reviews are not based upon empirical data.

**Table 7:** Design of studies within active citizenship impacts ^1^.

Design	Community Benefits	Integration and Inclusion	Volunteering	Bonding Capital
Overall	9	12	1	5
Meta	0	0	0	0
RCT	0	0	0	0
Case Control	0	0	0	0
Longitudinal	2	3	0	0
Cross-sectional	0	0	0	1
Qualitative data ^2^	5	6	0	3
Literature reviews ^3^	2	3	1	1

^1^ Studies may refer to one or more impacts, therefore the total sum can be higher than the number of studies within the overall impact category; ^2^ Qualitative studies include evaluations of case studies or interventions with qualitative research methods and expert opinion based on field experience; ^3^ Literature reviews are not based upon empirical data.

**Table 8:** Design of studies within impacts on crime reduction and (anti-)social behavior ^1^.

Design	Prosocial Behavior	Crime Reduction
Overall	7	4
Meta	0	0
RCT	0	0
Case Control	1	0
Longitudinal	1	1
Cross-sectional	0	0
Qualitative data ^2^	4	1
Literature reviews ^3^	1	2

^1^ Studies may refer to one or more impacts, therefore the total sum can be higher than the number of studies within the overall impact category; ^2^ Qualitative studies include evaluations of case studies or interventions with qualitative research methods and expert opinion based on field experience; ^3^ Literature reviews are not based upon empirical data.

**Table 9:** Design of evidence within additional impacts ^1^.

Design	Lifetime Physical Activity	Accessibility	Other Effects
Overall	19	4	8
Meta	1	0	0
RCT	1	0	0
Case Control	2	0	1
Longitudinal	3	0	1
Cross-sectional	2	0	1
Qualitative data ^2^	7	2	2
Literature reviews ^3^	3	2	3

^1^ Studies may refer to one or more impacts, therefore the total sum can be higher than the number of studies within the overall impact category; ^2^ Qualitative studies include evaluations of case studies or interventions with qualitative research methods and expert opinion based on field experience; ^3^ Literature reviews are not based upon empirical data.

## Supplementary Materials

The following are available online at https://www.mdpi.com/1660-4601/16/6/937/s1, Table S1: List of included activities, Table S2: List of selected studies, Table S3: Origin of selected studies.
