# Supplementary material for: Benefits of Outdoor Sports for Society. A Systematic Literature Review and Reflections on Evidence
Source: Int J Environ Res Public Health. 2019 Mar 15;16(6):937. doi: 10.3390/ijerph16060937 (PMC6466442; doi:10.3390/ijerph16060937)
Supplement: Supplementary file 1 [file ijerph-16-00937-s001.zip › IJERPH_S1.pdf]

S1: Range of outdoor sports and activities inside and outside the definition (non-exhaustive)

**Clearly within the definition**	**Maybe in the definition** (Depending on context)	**Outside the definition**
Hiking, Trekking	Outdoor education	Walking (strolling)
(Trail) Running, Nordic walking	Friluftsliv	Taking the dog out
Triathlon, Adventure racing	Survival training	Camping, Bird watching
Orienteering	Wilderness	Beach Volley, Football, Tennis
Cycling/Biking, MTB, Road biking	Alpine skiing, snowboarding and other snow sports on slopes	Skateboarding/Inline skating
Horse riding	Fishing/Angling	Spinning
Ski touring, cross country skiing/nordic skiing	Rope course activities	Motocross, Squad
Snow shoeing, Freeride /off piste		Jet skiing/Motor Boating, Wakeboarding/Water skiing
Swimming (in a natural element)		Golf, Minigolf
Canoeing/Kayaking, Rowing, Sailing, (Wind)Surfing, Canyoning, Diving, Coasteering, Rafting, Kite surfing		Shooting/Archery
Scrambling, Mountain and Rock climbing, Bouldering, Ice climbing, Mountaineering		Hunting
Caving		
Paragliding		
