# Supplementary material for: Benefits of Outdoor Sports for Society. A Systematic Literature Review and Reflections on Evidence
Source: Int J Environ Res Public Health. 2019 Mar 15;16(6):937. doi: 10.3390/ijerph16060937 (PMC6466442; doi:10.3390/ijerph16060937)
Supplement: Supplementary file 1 [file ijerph-16-00937-s001.zip › IJERPH_S2.pdf]

S2: List of selected studies (n=133)

Ref. Number	Full Reference	Title in English	Meth. Code*	Country	Participants			Study Description	Type of Sport	Core Outcomes	Key Findings
					Number	Age	Population				
3	Thompson Coon, J., Boddy, K., Stein, K., Whear, R., Barton J. & Depledge. M. H. (2011). Does Participating in Physical Activity in Outdoor Natural Environments Have a Greater Effect on Physical and Mental Wellbeing than Physical Activity Indoors? A Systematic Review. *Environmental Science & Technology* , 45(5), pp.1761–1772.	Does Participating in Physical Activity in Outdoor Natural Environments Have a Greater Effect on Physical and Mental Wellbeing than Physical Activity Indoors? A Systematic Review.	A	UK	833	adults	Most participants (523 adults) were young students	A systematic review of the comparative effects of participating in indoor and outdoor activity including 11 papers in total. All interventions consisted of a single episode of walking or running indoors with the same activity at a similar level conducted outdoors on a separate occasion	walking and running	1) feelings of revitalization and positive engagement<br>2) decrease in tension, confusion, anger and depression<br>3) decreased feelings of calmness (negative)<br>4) long-term adherence to physical activity	Most trials showed some improvement in mental wellbeing on one or other of the outcome measures. As a summary the authors conclude that exercising in natural environments was associated with greater feelings of revitalization and positive engagement, decreases in tension, confusion, anger, and depression, and increased energy. Participants also reported greater enjoyment and satisfaction with outdoor activity and declared a greater intent to repeat the activity at a later date. However, as a possible negative effect, the results suggest that feelings of calmness may be decreased following outdoor exercise.
5	Dickson, T.J., Gray, T. & Mann, K. (2008). *Australian Outdoor Adventure Activity Benefits Catalogue.* Centre for Tourism Research, University of Canberra.	Australian Outdoor Adventure Activity Benefits Catalogue.	A	AU	n/a	n/a	n/a	Literature review that gathers evidence from Australia and New Zealand on benefits of outdoor adventure activities. This includes 117 studies coming from various fields of research such as education, recreation, leisure, tourism, sport, adult learning, health, and therapy. Documents have been included from 1995 till 2008.	outdoor adventure activities such as hiking, mountain biking, climbing, abseiling, kayaking and canoeing, as well as Outward Bound and wilderness programs	1) Improved health, wellbeing and quality of life<br>2) positive effects in psycho-social, psychological, physical and spiritual domains, particularly with regards to interpersonal and intrapersonal skills development: increased self-efficacy, intellectual flexibility, self-confidence and problem solving skills, emotional control and academic achievement, communication, assertion, and inner strength, relationship building and improved long-term relationships with others<br>3) environmental awareness and stewardship<br>4) community benefits like heightened community identity, bonding families and communities<br>5) therapeutic tool for disabled, disordered adolescents or at-risk youth (intellectual flexibility, emotional control, social competence, achievement motivation, active initiative, and self-confidence, improved problem behavior, greater self-actualisation, decreased hopelessness, transitory increase in sense of wellbeing, and reduced recidivism)	The authors emphasize the unique opportunities provided by outdoor adventure activities within the natural and social environments offered The main benefits are seen in the development of interpersonal and intrapersonal skills. Benefits are shown to be evident in the fields of psycho-social, psychological, physical and spiritual domains, particularly with regards to developing self-efficacy, self-confidence and problem solving skills, intellectual flexibility, communication, and relationship building. Benefits for the natural environment were less directly evidenced. However, the authors point to indirect impacts like development of environmental awareness and stewardship, as well as the development of more nurturing individuals and communities. The literature review also highlights studies and programs that use outdoor activities as a therapeutic tool, as for disabled (life effectiveness was increased in intellectual flexibility, emotional control, social competence, achievement motivation, active initiative, and self-confidence), for disordered adolescents (improved problem behavior) or for at-risk youth (greater self-actualisation, decreased hopelessness, transitory increase in sense of wellbeing, and reduced recidivism).
8	Duncan, M.J., Clarke, N.D., Birch, S.L., Tallis, J., Hankey, J., Bryant, E. & Eyre, E.L.J. (2014). The Effect of Green Exercise on Blood Pressure, Heart Rate and Mood State in Primary School Children. *International Journal of Environmental Research and Public Health.* 11(4), pp.3678-3688	The Effect of Green Exercise on Blood Pressure, Heart Rate and Mood State in Primary School Children.	B	UK	14	Mean age = 10 years (SD = ± 1)	primary school children	Explorative study to examine the effect on blood pressure, heart rate and mood state responses in primary school children of moderate intensity cycling whilst viewing a green environment compared to exercise alone	green exercise - cycling	1) lower blood pressure	The results of the study suggest an augmented post exercise hypotensive effect for children following green exercise compared to exercise alone. This could be shown for a lower systolic blood pressure following green exercise.
9	Pretty, J., Peacock, J., Sellens, M. & Griffin, M. (2005). The mental and physical health outcomes of green exercise. *International Journal of Environmental Health Research.* 15(5), pp.319-337.	The mental and physical health outcomes of green exercise.	B	UK	100 (including controls)	18 – 60 years, with a mean of 24.6	adults	Experimental study on the synergistic effects of exercise and green environments; 5 groups were tested on a treadmill with exposure to rural and urban, pleasant and unpleasant scenes, and compared to exercise only measurements	green exercise	1) health benefits such as improvements in blood pressure, self-esteem and mood	The exercise alone group showed significant improvements in blood pressure, self-esteem and had a positive effect on 4 out of 6 mood measures. However rural and urban pleasant scenes resulted in even significantly greater positive effect on self-esteem. The authors therefore suggest a synergistic effect of exercise and green environments.<br>In contrast, exposure to unpleasant scenes seem to reduce the positive effects of exercising with the greatest decrease in rural unpleasant scenes.

Ref. Number	Full Reference	Title in English	Meth. Code*	Country	Participants			Study Description	Type of Sport	Core Outcomes	Key Findings
					Number	Age	Population				
10	Hartig, T., Evans, G.W., Jamner, L.D., Davis, D.S., & Garling, T. (2003). Tracking restoration in natural and urban field settings. *Journal of Environmental Psychology* , 23(2), pp.109-123.	Tracking restoration in natural and urban field settings.	B	US	112	young adults	young adults living in cities	Comparison of psychophysiological stress recovery and directed attention restoration in natural and urban field settings (the experimental design crossed an environmental treatment condition (natural, urban) with a pretreatment task condition (task, no-task)	walking in a nature reserve	1) physical and psychological restoration of people living in cities<br>2) blood pressure change, stress reduction, increase in positive affect and decrease in anger	Results suggest that walking in a nature reserve fostered blood pressure change that indicated greater stress reduction than afforded by walking in urban surroundings. Furthermore, positive affect increased and anger decreased in the nature reserve by the end of the walk while the opposite pattern emerged in the urban environment. The results speak to widely held beliefs that natural surroundings and green exercise can support the physical and psychological restoration of people living in cities.
11	Izenstark, D., Oswald, R.F., Holman, E.G. & Mendez, S.N. (2016). Rural, Low-Income Mothers' Use of Family-Based Nature Activities to Promote Family Health. *Journal of Leisure Research* , 48(2), pp.134–155.	Rural, Low-Income Mothers' Use of Family-Based Nature Activities to Promote Family Health.	F	US	85	19 to 59 years	low income families	Evaluation on the mothers engagement in family-based nature activities to promote physical, psychological, and social health	outdoor recreation with family (in parks or natural areas) including walking, hiking etc.	1) low-cost opportunity for health promotion in low-income families	The findings from this study demonstrate how mothers from low-income families use nature-based activities to promote the physical, psychological, and social health of each family member and the family as a whole within the context of rural poverty. Nature-based family activities serve as a vehicle to provide individual and family health benefits.
12	Liu, M.-h. & Liu, Z.-q. (2010). Effect of Winter Swimming on the Cardiovascular Function of the Seniors and Middle-age Population. *Journal of Beijing Sport University,* 33(4), pp.63-65.	Effect of Winter Swimming on the Cardiovascular Function of the Seniors and Middle-age Population.	E	CN	n/i	middle aged to seniors	middle aged and seniors	Cross-sectional study on the benefits of winter swimming on the cardiovasular function; one time of measurement and comparison to prevalence data from literature	winter swimming	1) physical health benefits on cardiovascular function<br>2) positive changes in the effect of blood hydromechanics, decreases blood fat, and increases the density of high-density fatty protein	The results shows that winter swimming improves the cardiovascular function of senior and middle-aged swimmers. Winter swimming also leads to changes in the effect of blood hydromechanics, decreases blood fat, and increases the density of high-density fatty protein.
13	Stoeppler, R. & Tillmann, V. (2004). '"Alle in einem Boot" Rudern als aktive Freizeitgestaltung bei Menschen mit geistiger Behinderung. *Praxis der Psychomotorik,* 29(3), pp.209-213.	"All in the same boat": rowing as an active form of leisure for people with mental retardation.	F	DE	n/a	n/a	people with mental retardation	Description of a recreational rowing program for people with mental retardation	rowing	1) physical health benefits<br>2) inclusion of people with mental disability<br>3) social group development<br>4) fast learning achievements	The authors give narrative insights on the benefits of an inclusive rowing programme that is focused on people with mental disabilities. Besides the well-known health benefits gained from physical activity (e.g. improved cardiovascular and pulmonary function, low injury rate), rowing is presented as a sport with fast learning achievements and with the opportunity for a heterogenous team. It also includes positive effects of nature experience and social group development.
14	Stuhl, A. & Porter, H. (2015). Riding the Waves: Therapeutic Surfing to Improve Social Skills for Children with Autism. *Therapeutic Recreation Journal,* 49(3), pp.253-256.	Riding the Waves: Therapeutic Surfing to Improve Social Skills for Children with Autism.	H	US	unclear, between 11 and 121 per program	5 to 18 years	children with autism	Literature review including three different surf programs; surf camp programs lasted 2-days, 6 or 8 weeks, no control groups in the interventions	therapeutic surfing	1) improved physical health and emotional wellbeing<br>2) improved social skills and competences (empathy, responsibility and engagement, communication, cooperation) as well as social trust<br>3) personal development (assertion, increased resilience, self-esteem, self-concept, self-control, self-confidence)<br>4) enjoyment and long-term physical engagement	Surfing is shown to significantly improve outcomes for assertion and empathy, responsibility and engagement, positive functioning, physical health, emotional wellbeing, resilience, self-esteem, vitality, friendship, social trust, and enjoyment in the outside environment. Additionally there were non-significant positive effects for social competence, social skills, and self-concept, communication, cooperation, responsibility, engagement, and self-control, self-confidence, confidence, self-esteem, well-being, motor skills, behavior, and re-engagement with school. Continued participation and peer relationships can be added as positive long-term benefits.
15	Zabriskie, R.B., Lundberg, N.R. & Groff, D.G. (2005). Quality of Life and Identity: The Benefits of a Community-Based Therapeutic Recreation and Adaptive Sports Program. *Therapeutic Recreation Journal,* 39(3), pp.176-191.	Quality of Life and Identity: The Benefits of a Community-Based Therapeutic Recreation and Adaptive Sports Program.	F	US	129	3 to 73 years; (M = 19,38 ± 16,59	individuals with disabilities	Survey evaluating the effects of a community-based therapeutic recreation and adaptive sports program for disabled on health, quality of life and community participation; 3 or 5 weeks program	horse riding, alpine skiing	1) inclusion of individuals with disabilities<br>2) improved quality of life including overall health, quality of family life, and quality of social life as well as athletic identity	Participation in a community based therapeutic recreation and adapted sports program had a positive influence on various dimensions of quality of life for people with disabilities. It improved overall health, quality of family life, and quality of social life as well as athletic identity. Therefore adaptive sport programs such as horse riding and skiing can help to prevent poor health, reduced quality of life and limited community participation of disabled people.
16	Donatiello E., Dello Russo M., Formisano A., Lauria F., Nappo A., Reineke A., Sparano S., Barba G., Russo P. & Siani A. (2013). Physical activity, adiposity and urbanization level in children: results for the Italian cohort of the IDEFICS study. *Public Health I,* 27, pp.761-765.	Physical activity, adiposity and urbanization level in children: results for the Italian cohort of the IDEFICS study.	E	IT	1673	2-8 years MV 6.1 SD 1.7	pre-school and school-aged children	Cross-sectional study to investigate the level of adiposity among children referring to the urbanization level and patterns of physical activity. The Italian cohort of the IDEFICS study is used.	outdoor activities including outdoor play and outdoor sport	1) time spent in outdoor activities as adiposities prevention for children (better effects than structured physical exercises)	Children who live in rural areas spent significantly more time in outdoor activities but participated in less structured physical activity (e.g. sport clubs) compared with children living in urban areas. Obesity assessed by the sum of skinfold thickness increased linearly from rural to urban areas. No significant differences in height, weight, BMI, waist circumference and prevalence of overweight/obesity were observed across the three areas. Although, weight, BMI, waist circumference and prevalence of overweight/obesity was higher in children living in urbar areas compared with children living in rural areas.

Ref. Number	Full Reference	Title in English	Meth. Code*	Country	Participants			Study Description	Type of Sport	Core Outcomes	Key Findings
					Number	Age	Population				
**17**	Doistua Nebreda, J. & Ried Luci, A. (2016). Ocio en la naturaleza como espacio de desarrollo juvenil. *Revista de psicología del deporte,* 25(4), pp.39-44.	Leisure in Nature as a space for youth development.	E	ES	1750 (247 outdoor activities / 1503 other activities)	16 to 25 years	youth (students in post compulsory education)	Analysis of main leisure activity and the relation to its benefits	outdoor sports in general	1) positive implications in youth development.<br>2) benefits in physical, emotional, cognitive, behavioural and social levels<br>3) positive effects in the level of responsibility and management	The main activity of leisure in nature has a significant positive influence on responsibility, management and in the general feeling of participants, in terms of physical, emotional, cognitive, behavioural and social levels.
**18**	Andre, E. K., Williams, N., Schwartz, F. & Bullard, C. (2017). Benefits of Campus Outdoor Recreation Programs: A Review of the Literature. *Journal of Outdoor Recreation, Education & Leadership,* 9(1), pp.15-25.	Benefits of Campus Outdoor Recreation Programs: A Review of the Literature.	H	US	n/a	n/a	young people (University and college students)	Literature review on the benefits of outdoor camps in universities and colleges	campus outdoor recreation	1) positive effects on student recruitment, retention, and satisfaction<br>2) increased academic success, smoother transitions to college<br>3) improved mental and physical health, lower levels of stress and anxiety<br>4) better and more numerous social connections and better intra and interpersonal skills<br>5) improved environmental sensitivity, and connectedness to nature and to place	The article gives a summary and confirms the existence of the range of benefits of campus based outdoor recreation for young people: increased academic success, smoother transitions to college, better mental and physical health, lower levels of stress and anxiety, better and more numerous social connections, better intra- and interpersonal skills, increased environmental sensitivity, and better connectedness to nature and to place
**19**	Drăgoi, C. C. (2014). Study regarding the influence of hiking on certain health markers in middle school students. *Sport & Society,* 14, pp.36-43.	Study regarding the influence of hiking on certain health markers in middle school students.	E	RO	n/i	n/i	middle school students who perform regularly mountaineering activities	A sample of middle school students that practice mountaineering activities regularly has been compared to an inactive control group	hiking and mountaineering	1) positive effects on physiometric and somatometric health markers	Regular practice of hiking and mountaineering activities is associated with certain physiometric amd somatometric health markers in middle school students.
**20**	Rosa, P., Carvalhinho, L. & Soares, J. (2017). O Desporto de Natureza e o Desenvolvimento Sustentável: Prespectivas de Desenvolvimento e Governança. *Revista de Educação Física da UFRGS, Movimento,* 23(1), pp.419-436.	Nature-based sports and sustainable development: Perspectives of development and governance.	H	PT	n/a	n/a	n/a	Literature review on the relation of nature sports with the economy, the environment and its role on the society, concluding with a critical reflexion on the concept of nature sports' sustainability.	scuba diving, climbing; surf, canoeing; sailing, rowing; swimming, nature sports in general.	1) promote ecological awareness<br>2) positive influence on health, quality of life and life satisfaction<br>3) potential to increase social inclusion among children and to prevent delinquency in youth<br>4) potential negative impact on the natural environment	Nature Sports revealed signs of a real contribution for rural economies and in some cases relevant in regional or national economies. In environmental terms it would not be wise to neglect the potential negative impact of nature sports in the nature environment, although nature sports can be a good way to build ecological awareness among people thereby decreasing the negative effects of actvities. The authors conclude that nature sports can contribute to the general development of our society with an education potential and associated.physical and mental benefits.
**21**	Oftedal, A. & Schneider, I. (2013). Outdoor Recreation Availability, physical activity, and health outcomes: County-level analysis in Minnesota. *Journal of Park and Recreation Administration,* 31(1), pp.34-56.	Outdoor Recreation Availability, Physical Activity, and Health Outcomes: County-Level Analysis in Minnesota	E	US	n/a	n/a	n/a	Macro-level study of the connection between the availability (supply) of outdoor recreation, physical activity and health by developing a typology of recreation opportunities and analysis a wide range of health outcomes	outdoor recreation	1) parkland and nonmotorized trails are significantly associated with increased physical activity and lower obesity rates	The results show the relationship between health and outdoor recreation supply were mixed. Results suggest that parkland and nonmotorized trails are significantly and positively associated with increased physical activity and lower obesity rates. However other health asociations remained insignificant. Results suggest that availability of varios different types of recreation opportunities can produce different types of benefits.
**22**	Pierskalla, C. D., Lee, M. A., Stein, T. V., Anderson, D. H. & Nickerson, R. (2004). Understanding relationships among recreation opportunieis: A meta analysis of nine studies. *Leisure Sciences,* 26(2), pp.163-180.	Understanding Relationships Among Recreation Opportunities: A Meta Analysis of Nine Studies.	F	US	n/a	n/a	n/a	Data from nine benefits-based management pilot-studies were collected, analysed and combined using meta-analysis techniques	outdoor recreation	1) increased happiness<br>2) physical fitness<br>3) personal development<br>4) self confidence<br>5) independence<br>6) spiritual strength	The attainment of benefits such as keeping / getting physically fit, feeling happier, improving skills and abilities, increased self confidence, a greater sense of independence and feeling stronger spiritually were quantified. It was noted that not all of the benefits came from specific activities or inputs by recreational managers.
**23**	Rosenberger, R. S., Bergerson, T. R. & Kline, J. D. (2009). Macro-Linkages between Health and Outdoor Recreation: The Role of Parks and Recreation Providers. *Journal of Park and Recreation Administration,* 27(3), pp.8-20.	Macro-Linkages between Health and Outdoor Recreation: The Role of Parks and Recreation Providers.	E	US	n/a	n/a	n/a	Using a macro-level model to aggregate secondary data reflecting health status indicators (physical activity levels and weight status) and recreation supply and demand to assess direct and indirect relationships	outdoor recreation including open space team sports as well as trail-related activities (e.g. hiking, jogging, cycling) and off-trail outdoor sports	1) links between adult physical activity, overweight, obesity, and recreation supply and demand<br>2) reduces obesity<br>3) activates sedentary, non-active people	The prevalence of hiking and urban trails is associated with higher proportions of physically active adults. Parks and recreation providers can play a significant role in the health and wellbeing of residents by providing infrastructure to support activity.

Ref. Number	Full Reference	Title in English	Meth. Code*	Country	Participants			Study Description	Type of Sport	Core Outcomes	Key Findings
					Number	Age	Population				
**24**	Hanson, S. & Jones, A. (2015). Is there benefit that walking groups have health benefits? A systematic review and meta-analysis. *British Journal of Sports Medicine,* (0), pp.0-7	Is there benefit that walking groups have health benefits? A systematic review and meta-analysis.	A	UK	1843 from 42 studies	mean age of 58	Adults (19+) involved in group walking outdoors	Systematic review and meta-analysis of walking group interventions (RCT and CT included) looking at physiological, psychological and well-being outcomes pre and post intervention.	Outdoor walking groups. (occasionally indoors due to weather). Majority of studies (75%) were below international moderate activity guidelines.	1) decreased systolic and diastolic blood pressure<br>2) decreased resting heart rate<br>3) decreased body mass index<br>4) decreased total cholesterol<br>5) decreased body fat<br>6) increased V02 Max<br>7) decreased depression	Forty-two studies were identified involving 1843 participants. Walking groups have wide ranging health benefits. They found statistically significant reductions in mean difference for systolic blood pressure, diastolic blood pressure, RHR, body mass index, total cholesterol and statisically signficant mean increases in VO2 Max and reduction in depression score. No adverse side effects. Walking groups are effective and safe with good adherence and wide-ranging health benefits. They could be a promising intervention as an adjunct to other healthcare or as a proactive health-promoting activity.
**25**	Roberson, D. N. & Babic, V. (2009). Remedy for modernity: experiences of walkers and hikers on Medvednica Mountain. *Leisure Studies,* 28(1), pp.105-112.	Remedy for modernity: experiences of walkers and hikers on Medvednica Mountain.	F	HR	122	n/a	n/a	Qualitative research to assess the motivations and benefits of walking / hiking amongst existing participants on Medvednica Mountain (Croatia) using informal interviews	walking, hiking	1) mental and physical health benefits<br>2) social interaction<br>3) affinity with nature and the outdoors<br>4) development of self-knowledge	The study highlights that walking and hiking can negate the impact of living in modern society by providing benefits relating to: affinity with nature and the outdoors, mental and physical benefits and interaction with others and development of self knowledge. Civic organisations should promote walking as a way of life.
**26**	Bachčevanski, S. (2016). Приспособления за адаптация на ветроходни яхти за хора с физически увреждания. *Sport & nauka* , 60(3), pp.120-127.	Adaptive equipment of sailboats for people with physical disabilities.	F	BG	n/a	n/a	people with physical disabilities	The aim of the study is to show, that sailing is a great sport for people with physical disabilities because it accommodates such a wide range of disabilities and with the use of adaptive equipment; individuals can function as independently as possible	sailing	1) inclusion of disabled<br>2) reduced risk of obesity	The author states that adaptive sailing is a great sport for people with physical disabilities, either as a recreational activity or a competitive high performance sport. It promotes freedom and independence, and is both accessible and inclusive. Participants are not segregated according to their physical abilities. Indeed, sailing is one of few sports that allows people with disabilities to directly compete against able-bodied participants.
**27**	Gešev, P. & Papazoglu, J. (2013). Динамика на развитието на силовите качества на 15-годишни ученици, практикуващи ветроходен спорт. *Sport & nauka* , 57(4), pp.97-105.	Dynamics study of the power development among 15 years old students practicing sailing.	C	GR	115	15	students	The study investigates the dynamics of power development between pupils practicing sailing in their free time and pupils not involved in sports training.	sailing	1) improvement of physical development<br>2) stimulates active lifestyles	The students who practiced sailing have better results in all survey indicators in comparison to the not active students. Sailing sport activities contribute to the improvement of the physical development of youth and stimulates active lifestyles.
**28**	Kux, S. & Wolfgang, H. (2014). *Health Benefits of Non-Motorized Outdoor Recreation: A Summary of Published Findings.* Burnaby, BC: School of Resource and Environmental Management, Simon Fraser University.	Health Benefits of Non-Motorized Outdoor Recreation: A Summary of Published Findings.	H	CA	n/a	n/a	n/a	Review on existing research on health benefits associated with non-motorized outdoor recreation activities	all types of outdoor recreation but particularly hiking, rock climbing and alpine skiing	1) OS and being in nature improves physical health (improved cardiovascular function and reduction in the risk of coronary artery disease, reduced body fat, improved hormone regulation and other metabolic adaptations, improved balance in the elderly, increases in the number of naturally generated stem cells, reductions in blood-bound “bad cholesterol”)<br>2) potential to attract participants and foster life-long hobbies	The study summarizes the evidence of physical health benefits attributed to non-motorized outdoor recreation in general and in particular for hiking, rock climbing and skiing. Several general health benefits such as improved cardiovascular function and reduction in the risk of coronary artery disease (-50% for hiking), reduced body fat, improved hormone regulation / production and other metabolic adaptations can be accrued through participation in any of the possible activities. For other health benefits like improved balance in the elderly, increases in the number of naturally generated stem cells, reductions in blood-bound “bad cholesterol” and others, evidence is linked to specific activities.
**29**	Verity, C. & Mackintosh, C. (2014). *Reconomics: The Economic Impact of Outdoor Recreation in the UK: The Evidence.* Sport and Recreation Alliance.	Reconomics: The Economic Impact of Outdoor Recreation in the UK: The Evidence.	G	UK	n/a	n/a	n/a	Synthesis of existing evidence on the economic value of outdoor recreation in the UK	various types of outdoor recreation activities including water sports, snowsports, cycling, horse riding and walking	1) reduce health related costs of physical inactivity<br>2) foster employment, skills and volunteering	The report collates data on participation, visitor expenditure, jobs and volunteers as well as health related costs and savings relating to outdoor recreation in the United Kingdom. The health costs of physical inactivity are estimated to be around £10 billion.
**30**	Bowles, B., Fleming, K., Fuller, K., Lankford, J. & Prinz, J. (2011). *Economic and Health Benefits of Bicycling in Iowa.* Coralville, Iowa: Iowa Bicycle Coalition.	Economic and Health Benefits of Bicycling in Iowa.	G	US	1090	n/i	cyclists in Iowa	study on the economic impact of bycicle riders and bicycle businesses and organisations in Iowa	commuter and recreational cycling	1) health benefits<br>2) healthcare cost savings	The study gives insights on profiles of the commuter and recreational cyclists (party and trip characteristics, spending, and demographic profiles) as well as health benefits and health cost savings from cycling in Iowa. Regarding health benefits, cycling commuters save the state $13.2 million in health care cost, those who cycle recreationally bring another $73.9 million in healthcare costs savings.

Ref. Number	Full Reference	Title in English	Meth. Code*	Country	Participants			Study Description	Type of Sport	Core Outcomes	Key Findings
					Number	Age	Population				
**31**	Morris, N. (2003). *Health, Well-Being, and Open Space: Literature Review.* Edinburgh College of Art and Heriot-Watt University, Edinburgh, UK: OPENspace, The Research Centre for Inclusive Access to Outdoor Environments.	Health, Well-Being, and Open Space: Literature Review.	H	UK	n/a	n/a	n/a	literature review on the benefits of open-air recreation and activities in outdoor spaces	outdoor recreation in general including walking, cycling, outward bound courses and others	1) increased physical health, increased life-span<br>2) enhanced mental and spiritual health<br>3) enhanced spiritual, sensory, and aesthetic awareness<br>4) ability to assert personal control and increased sensitivity to one's own well-being<br>5) enhanced personal and social communication skills, heightened social interaction<br>6) increased quality of life<br>7) enhanced connections between people and the natural environment<br>8) motivation and increased adherence to physical activity<br>9) lower rates of smoking and substance misuse	The literature review revealed that 5 key benefits from exposure to the natural environment to human health could be highlighted: 1) Enhanced personal and social communication skills, 2) Increased physical health and life-span, 3) Enhanced mental and spiritual health, 4) Enhanced spiritual, sensory, and aesthetic awareness, 5) Ability to assert personal control and increased sensitivity to one's own well-being.<br>This also includes increased quality of life, enhanced feelings of well-being, fewer symptoms of depression, lower rates of smoking and substance misuse, enhanced relaxation and refreshment, reduction of anxiety and stress levels; enhanced motivation and increased adherence to physical activity. Furthermore, outdoor recreation is also used as effective form of complementary therapy, for example for the treatment of mental illness
**32**	de Moor, Des. (2015). *Walking Works: Making the case to encourage greater uptake of walking as a physical activity and recognise the value and benefits of Walking for Health.* Ramblers and Macmillan Cancer Support: London, UK.	Walking Works: Making the case to encourage greater uptake of walking as a physical activity and recognise the value and benefits of Walking for Health.	H	UK	n/a	n/a	n/a	literature review on the health benefits of walking	walking	1) physical health benefits: reduced risk of high blood pressure, diabetes and coronary heart disease, stroke, colon and breast cancer, reduced cholesterol, Alzheimer's disease and expanded life expectancy<br>2) mental health benefits: cognitive function and less cognitive decline<br>3) opportunity for social contacts and relations<br>4) cost-effective, safe and accessible	The report summarizes the health and wellbeing benefits gained from walking. The authors state that this activity delivers all the benefits physical activity in general is discussed for, with the added benefit of being accessible to the majority of the population.<br>Health benefits discusssed include reduced risk of high blood pressure, diabetes, coronary heart disease, stroke, colon and breast cancer, cholesterol, Alzheimer's disease and expanded life expectancy. It also improves physical and mental health especially for older people, e.g. cognitive function and 12% less risk of cognitive decline in older people for every hour walked and improved control of blood sugar levels in older people at risk of developing type 2 diabetes. Furthermore, the report highlights the social aspects of group walks. Overall, walking is promoted as an inexpensive, safe and effective way of moving people and can be an answer to fighting inactivity.
**33**	Mapes, N. (2016). Green exercise and dementia. In Barton, J., Bragg, R., Wood, C. & Pretty, J.N. (Eds.). *Green Exercise: Linking Nature, Health and Well-Being* (pp.150-160). London: Routledge	Green exercise and dementia.	F	UK	n/a	n/a	n/a	Narrative description of various programs for people suffering from dementia; the study gives insights on expert estimations and statements of participants, family members and carers.	walking in nature, adventure holidays including activities such as sailing and white wate rafting	1) improving the physical and mental health and wellbeing of people with dementia<br>2) prevention and slowing down of the progression of dementia<br>3) increasing happiness and quality of life for people with dementia<br>4) social benefits like improved opportunities for people with dementia to maintain connections with nature and other people, interaction with people and places	Based on relevant literature and various qualitative statements from case studies the authors give a narrative insight on the benefits of green exercise for people with dementia. Those include physical benefits like better eating and sleeping patterns, better fitness and mobility and fewer falls, but also psychological, and wellbeing benefits like increased happiness, an improved emational state through reduced stress, agitation, anger, apathy and depression, and a higher self-esteem. Furthermore social benefits like improved interaction with other people, the reconnection with places and activities they loved and a sense of belonging helps patients to lead a better quality live.
**34**	Moore, S.C., Lee, I-M., Weiderpass, E., Campbell, P. T., Sampson, J.N., Kitahara, C.M. et al. (2016). Association of Leisure-Time Physical Activity With Risk of 26 Types of Cancer in 1.44 Million Adults. *JAMA Internal Medicine,* 176(6), pp.816-825. Published online	Association of Leisure-Time Physical Activity With Risk of 26 Types of Cancer in 1.44 Million Adults.	D	US	1.44 Million	adults, median age: 59 years [range 19-98]	US and European cohorts; 57% female; 186 932 cancers were included.	Data from 12 prospective US and European cohorts with self-reported physical activity (baseline, 1987-2004)	leisure-time physical activities including cycling, swimming etc.	1) lower risk of 13 types of cancer<br>2) higher risk for malignant melanoma and prostate cancer	High levels of leisure-time physical activity were associated with lower risks of 13 cancer types. This included esophageal (- 42%), liver (- 27%), lung (-26%), kidney (-23%), stomach (-22%), endometrial (-21%), myeloid leukemia (-20%), myeloma (-17%), colon (-16%), head and neck (-15%), rectal (-13%), bladder (-13%) and breast cancer (-10%). Leisure-time physical activity was associated with lower risks of many cancer types regardless of body size or smoking history. Leisure-time physical activity however is also associated with higher risks of malignant melanoma and prostate cancer
**35**	Blond, K., Rasmussen, M., Østergaard, L. & Grøntved, A. (2016). Prospective Study of Bicycling and Risk of Coronary Heart Disease in Danish Men and Women. *Circulation,* 134(18), pp.1409-1411.	Prospective Study of Bicycling and Risk of Coronary Heart Disease in Danish Men and Women.	D	DK	53.723	50 - 65 years	elderly	Prospective study that evaluates the relationship between cycling habits and risk of CHD. Researchers tracked 53 723 Danes 50 to 65 years of age at recruitment in 1993 to 1997 from the prospective cohort study, "Diet, Cancer, and Health" for 20 years.	Cycling	1) decreased risk of heart disease	People who frequently bike to work or in their free time have a decreased risk of heart disease (11 to 18 percent fewer heart attacks). Participants who started and then maintained active biking had a 26 percent lower risk of developing coronary artery disease.

Ref. Number	Full Reference	Title in English	Meth. Code*	Country	Participants			Study Description	Type of Sport	Core Outcomes	Key Findings
					Number	Age	Population				
**36**	Thijssen, D.H.J., de Groot, P.C.E., Smits, P. & Hopman, M.T.E. (2007). Vascular adaptations to 8-week cycling training in older men. *Acta Physiologica* . 190(3), pp.221-228.	Vascular adaptations to 8-week cycling training in older men.	D	NL	8	70 ± 3 years	older men	Effects of an 8 weeks endurance cycling training on vascular adaptations in sedentary, older men; pre-post measurements	cycling	1) improve in cardiovascular function	The eight weeks of cycle training that a group of older healthy but sedentary men participated in, altered functional and structural characteristics of the lower extremity vasculature. However, no changes were found for the conduit arteries in the non-trained areas. As a conclusion, endurance training induces vascular adaptations in older men rapidly, and may help to prevent age-related changes and cardiovascular pathology.
**37**	Niederseer, D., Ledl-Kurkowski, E., Kvita, K., Patsch, W., Dela, F., Mueller, E., et al. (2011). Salzburg Skiing for the Elderly Study: changes in cardiovascular risk factors through skiing in the elderly. *Scandinavian Journal of Medicine & Science in Sports,* 21**,** pp.47-55.	Salzburg Skiing for the Elderly Study: changes in cardiovascular risk factors through skiing in the elderly.	B	AT	42	60 to 76 years	erderly	Intervention program with randomized control group design including 28.5 days of guided skiing during 12 weeks. Cardiovascular risk factors (CVRF) were assessed pre and post treatment.	alpine skiing	1) health benefits in cardiovascular system	The authors analysed alpine skiing as a treatment of the modifiable cardiovascular risk factors in order to decrease the risk to suffer from stroke or myocardial infarction. No cardiovascular event occurred within a total of 795.1 h of skiing while exercise capacity and oxygen consumption increased, and body fat decreased significantly. However blood pressure, blood lipids (the fatty substances found in the blood, including cholesterol), heart rate and everyday physical activity remained essentially unchanged. The authors conclude that alpine skiing in the elderly is safe with respect to cardiovascular events, and improves some, but not all cardiovascular risk factors.
**38**	Heggie, T. W., & Heggie, T. M. (2012). Dead men hiking: Case studies from the american wilderness. In *Medicina sportiva : official journal of Polish Society of Sports Medicine* , 16(3), pp.118-121.	Dead men hiking: Case studies from the american wilderness.	F	US	4	21, 44, 51 and n.i.	n/a	Case studies from the American wilderness, investigates the factors that led to the death of four hikers.	hiking	1) low mortal risk	Environmental factors and factors related to acute bad judgment syndrome can contribute to the death of hikers. The authors come to the conclusion that all factors that lead to the death were preventable.
**39**	Wilson, I., McDermott, H., Munir, F., & Hogervorst, E. (2013). Injuries, ill-health and fatalities in white water rafting and white water paddling. In *Sports medicine* , 43(1), pp.65-75.	Injuries, ill-health and fatalities in white water rafting and white water paddling.	A	UK	n/a	n/a	n/a	Literature review to identify the types of injuries and ill-health in white water activities. Injury and fatality rates were assessed to establish the risk attributed to these activities.	white water (WW) activities such as paddling (canoeing and kayaking) and rafting	1) low injury rate	The shoulders and back are considered as the most vulnerable sites for injury in WW paddling, whereas injuries to the face and lower limbs are seen as most common in WW rafters. However the authors conclude that injury rates can be estimated as low in WW activities.
**40**	Ruedl, G., Schranz, A., Fink, C., Woldrich, T., Sommersacher, R., Nachbauer, W., & Burtscher, M. (2009). Knieverletzungen bei Frauen im Freizeitskilauf: Risikofaktoren und Präventivmaßnahmen im Überblick. In *Deutsche Zeitschrift für Sportmedizin* , 60(11), pp.345-349.	Knee injuries in female recreational alpine skiing	H	DE	n/a	n/a	women	Literature overview of internal and external risk factors and prevention of knee injuries in female recreational alpine skiing	alpine skiing	1) low injury rate	The average injury rate in alpine skiing in general is relatively low (<2.0 injured persons per 1000 skier days). 50 percent of serious knee injuries in alpine skiing affect the anterior cruciate ligament (ACL). However, female recreational skiers have a threefold higher risk to sustain an ACL injury than male skiers. The authors state internal factors like higher age, the preovulatory phase and a low fitness level as a reason to put female skiers at an increased risk for ACL injury. Additionally, external risk factors such as skiing equipment and environmental factors can lead to a knee injury.
**41**	White, M.P., Elliott, L.R., Taylor, T., Wheeler, B.W., Spencer, A., Bone, A., et al. (2016). Recreational physical activity in natural environments and implications for health: A population based cross-sectional study in England. *Preventive Medicine,* 91, pp.383-388.	Recreational physical activity in natural environments and implications for health: A population based cross-sectional study in England.	E	UK	280790	adults	English adults	Cross-sectional analysis of six waves (2009/10–2014/5) of the nationally representative, Monitor of Engagement with the Natural Environment survey	active visits to natural environments including swimming and other watersports, running, cycling, horse riding	1) extends life expectancy<br>2) appealing and sustainable for all of the population	The study estimates the total annual amount of physical activity associated with recreational visits to natural environments by adults and gives implications for population health. The results show that English adults practice 3.2 million active visits to natural environments per year. Those can furthermore be associated with an extended life expectancy of 109,164 Quality Adjusted Life Years (QALY) annually. As calculations of the social value of a QALY are estimated to be £20,000, the annual value of these green active visits is approximately £2.18 billion.
**42**	Kelly, P., Kahlmeier, S., Götschi, T., Orsini, N.,Richards, J., Roberts, N., Scarborough, P. & Foster, C. (2014). Systematic review and meta-analysis of reduction in all-cause mortality from walking and cycling and shape of dose response relationship. *International Journal of Behavioral Nutrition and Physical Activity. 11(1*), 132.	Systematic review and meta-analysis of reduction in all-cause mortality from walking and cycling and shape of dose response relationship.	A	UK	280,000 participants for walking and 187,000 individuals for cycling	n/a	n/a	Systematic review to investigate the beneficial effects of regular walking and cycling on all-cause -mortality	walking and cycling	1) reduced risk of all-cause mortality	As a result of the systematic review the authors come to the conclusion that walking and cycling have population-level health benefits reducing the all-cause mortality risk by an average of 11 and 10% respectively.

Ref. Number	Full Reference	Title in English	Meth. Code*	Country	Participants			Study Description	Type of Sport	Core Outcomes	Key Findings
					Number	Age	Population				
43	Hasler, R. M., Huttner, H. E., Keel, M. J. B., Durrer, B., Zimmermann, H., Exadaktylos, A K. and Benneker, L. M. (2012). Spinal and pelvic injuries in airborne sports: A retrospective analysis from a major Swiss trauma centre. *Injury,* 43(4), pp.440-445.	Spinal and pelvic injuries in airborne sports: A retrospective analysis from a major Swiss trauma centre.	E	CH	181	median age 37	Spinal and pelvic injuries admitted to Level 1 trauma in the Swiss Alps, 89% were male.	Retrospective analysis of all airborne sports-associated spinal and pelvic injuries admitted between March 2000 - October 2009 were compared with spinal and pelvic fractures in the general trauma population using multiple logistic regression analysis.	BASE jumping, paragliding, parachuting, delta-gliding, speedflying, skysurfing.	1) higher rate of serious spinal and pelvic injuries due to airborne sports	The authors found a higher rate of serious spinal and pelvic injuries caused by airborne sports. There is a 21-fold higher odds ratio for spino-pelvic dissociation fractures in paragliders than the general trauma population. Age and gender were not significant. 49.2% patients sustained spinal fractures. Spinal fractures were classified using the magerl classification (Type A, B, C). 91.5% had type A fracture, 5.3% had Type C and 3.2% had Type B. With pelvic fractures, level L1 was most commonly affected (35.1%) Out of 17 patients who suffered a pelvic injury, 41.2 were Type C, and 29.4% were each Type A and B.
44	Aleman, K. B. & Meyers, M. C. (2010). Mountain Biking Injuries in Children and Adolescents. *Sports Medicine,* 40(1), pp.77-90.	Mountain Biking Injuries in Children and Adolescents.	H	US	n/a	n/a	n/a	Discussion paper of Mountain biking and associated injuries. Causality, risk factors and prevention methods are also discussed.	mountain biking - downhill, cross country	1) specific paediatric mountain biking injuries	Mountain biking can result in: cranial and thoraco-abdominal trauma, head and neck trauma, concussions, neurological sequelae, limb injuries and muscle strains. Recommendations are given on minimising the severity of injuries and maximising performance.
45	Crane, M., Rissel, C., Standen, C. & Greaves, S. (2014). Associations between the frequency of cycling and domains of quality of life. *Health Promotion Journal of Australia,* 25(3), pp.182-185.	Associations between the frequency of cycling and domains of quality of life.	E	AU	846	18 to 55 years	men and women, residents of Sydney	Examination of the association between domains of quality of life and the frequency of cycling by men and women including a cross-sectional survey of 846 healthy adults in Sydney	cycling	1) increased physical quality of life and psychological wellbeing in men	Results suggest that cycling offers physical and psychological quality of life benefits for men whereas no significant effects were observed for women. There was no relation between cycling and environment and social quality of life. In order to increase physical quality of life, cycling should be practiced at least weekly. For benefits associated with psychological wellbeing, any cycling, no matter what frequency, led to positive results.
46	Puett, R., Teas, J., Espana-Romero, V., Garcia Artero, E., Duck-chul, L., Baruth, M., Sui, X., Montresor-Lopez, J. & Blair, S. N. (2014). Physical Activity: Does Environment Make a Difference for Tension, Stress, Emotional Outlook, and Perceptions of Health Status? *Journal of Physical Activity and Health,* 11, pp.1103-1511.	Physical Activity: Does Environment Make a Difference for Tension, Stress, Emotional Outlook, and Perceptions of Health Status?	E	US	11.649	Over 20 years	n/a	Cross-sectional data collected via an observational epidemiology study. Large-scale research combining questionnaire data with clinical evaluation of participants involved in a Aerobics Centre Longitudinal Study (ACLS).	Indoor walking, jogging, running, cycling activitiy (treadmill or stationary bike) or outdoor activity (walking, jogging, running or cycling outdoors)	1) better stress management<br>2) protective effects for tension and poor emotional outlook<br>3) better overall health perceptions	The addition of outdoor activity for participants may be linked with better stress management, outlook and health perceptions for more active populations, whereas indoor physical activity may be more important for low active populations. One of the strongest findings was a possible effect of combined physical activity environment (indoor + outdoor) with respect to poor emotional outlook in women. Exercise in any of the 3 environments (indoor, outdoor or combined) was protective for tension, stress management, poor emotional outlook and poor overall health perceptions - with combined or outdoor envirnoments most effective.
47	Minello, K. & Nixon, D. (2017). 'Hope I never stop': older men and their two-wheeled love affairs. *Annals of Leisure Research,* 20(1), pp.75-95.	'Hope I never stop': older men and their two-wheeled love affairs.	F	CA	12	54 to 82 years	older men	Based on a phenomenological approach this study illuminates the meanings and experiences of road cycling in a group of older men, specifically in the context of ageing	road cycling	1) activity with few limitations to participation<br>2) healthy and happy ageing	The study highlights cycling as a physical activity with few limitations to participation and a conduit to exercise and well-being. Besides enjoying the activity itself, feeling fit and well, the participants reported on experiences of self, camaderie and embracing ageing. For that purpose, cycling is also discribed as an opportunity for older men to resist and reconceptualize society's tragic decline in the discourse about ageing.
48	Müller, E., Gimpl, M., Kirchner, S., Kröll, J., Jahnel, R., Niebauer, J., et al. (2011). Salzburg Skiing for the Elderly Study: influence of alpine skiing on aerobic capacity, strength, power, and balance. *Scandinavian Journal of Medicine & Science in Sports,* 21, pp.9-22.	Salzburg Skiing for the Elderly Study: influence of alpine skiing on aerobic capacity, strength, power, and balance.	B	AT	47	60 to 76 years	elderly	Intervention programme with randomized control group design including 28.5 days of guided skiing during 12 weeks. Aerobic capacity, leg power, strength and postural stability were tested before, immediately after, and 10 weeks after the intervention phase	alpine skiing	1) healthy ageing<br>2) maintain physical performance and therewith mobility and quality of life in elderly	A 12 week skiing intervention led to a significant increase in aerobic capacity, leg muscle power, and strength in older individuals. Aerobic capacity improved by 7,2% and jump height by 6% while in the control group, the former stayed constant and the latter decreased by 11,7% . Leg strength increased by 16% in the intervention and 7% in the control group. The authors suggest that alpine skiing can be a suitable activity to maintain physical performance and therefore mobility and quality of life or at least reduce negative changes in neuromuscular and cardiovascular systems due to the ageing process.
49	Dan, S. (2007). Effect of Winter Outdoor Swimming on Old people's Microcirculation and Hemorheological Changes. *Journal of Beijing Sport University,* 30(9), pp.1231-1233.	Effect of Winter Outdoor Swimming on Old people's Microcirculation and Hemorheological Changes.	C	CN	n/i	n/i	older people	Study on the influence of winter outdoor swimming on microcirculation and hemorheological changes in older people; Comparisons were made between people engaged in winter swimming exercise and a control group that rarely exercised	winter outdoor swimming	1) improved microcirculation and hemorheological change in older people	The authors suggest that winter outdoor swimming can enhance microcirculation and improve hemorheological change in older people.

Ref. Number	Full Reference	Title in English	Meth. Code*	Country	Participants			Study Description	Type of Sport	Core Outcomes	Key Findings
					Number	Age	Population				
**50**	De Rui M., Toffanello E. D., Veronese N., Zambon S., Bolzetta F., Sartori L., Musacchio E., Corti M. C., Baggio G., Crepaldi G., Perissinotto E., Manzato E., Sergi G. (2014). Vitamin D Deficiency and Leisure Time Activities in the Elderly: Are All Pastimes the Same? *PLOS ONE*, 9(4), e94805.	Vitamin D Deficiency and Leisure Time Activities in the Elderly: Are All Pastimes the Same?	E	IT	2349	> 65 years	well performing elderly persons who fullfiled certain criterias (e.g. standardized 6-minute walking test - 6MWT)	Study on the effect of different pasttimes of elderly people on the Vitamin D status described by serum 25OHD level. The sample consists of 2,349 community-dwelling elderly people living in the Progetto Veneto Anziani. Outdoor activities have been compared to indoor activities such as dancing and gym workouts.	outdoor activities: brisk walking, cycling, gardening and fishing	1) positive effect on 25OHD level (vitamin D status) in elderly persons	Elderly people (both genders) practicing outdoor activities had higher median serum 25OHD levels (which is an indicator for Vitamin D status) than those who did not participate.<br>Outdoor physical activities are not all equally beneficial in terms of vitamin D status as they are differing in terms of sun exposure. Activities like cycling or gardening for at least an hour a week may help to reduce the risk of vitamin D deficiency in well-performing elderly people. The authors advise that recommendations concerning hypovitaminosis D epidemic for elderly persons should not only be vitamin D supplementation, but also promote the benefits of outdoor activities.
**51**	Dalmay F., Bhalla D., Nicoletti A., Cabrera-Gomez J. A., Cabre P., Ruiz F., Druet-Cabanac M., Dumas M. & Preux P. M. (2010). Multiple sclerosis and solar exposure before the age of 15 years: case–control study in Cuba, Martinique and Sicily. *Multiple Sclerosis J*, 6(8), pp.899-908.	Multiple sclerosis and solar exposure before the age of 15 years: case–control study in Cuba, Martinique and Sicily.	C	IT	total: 551 (cases: 193 Controls : 358)	15-75	population of the named islands which lifed there before the age of 15	Investigation on the protective role of childhood solar exposure on multiple sclerosis in residents of Cuba, Martinique and Sicily. Results are compared to a control group without neurological disorder that is matched for sex, age (5 years) and residence before age 15.	outdoor leisure activities in general; particularly water sports	1) sun exposure can prevent multiple sclerosis	The authors conclude that outdoor leisure activities in addition to sun exposure are connected to a reduced risk of multiple sclerosis.
**52**	Russo A., Semeraro F., Romano M. R., Matropasqua R., Dell'Omo R. & Castagliola C. (2014). Myopia onset and progression: can it be prevented? *Int Ophthalmol*, 34, pp.693-705.	Myopia onset and progression: can it be prevented?	H	IT	n/a	n/a	n/a	Literature review on the topic of myopia onset and progression including 123 studies. 26 studies are cited to demonstrate the positive effect of outdoor activities including outdoor sport on avoidance and slowing down the progress of myopia. 7 studies are explicitly examining the effect of outdoor activities. Among them are meta-analyses, systematic reviews and cohort studies, which suggests a robust data set.	outdoor activities including outdoor sport	1) time spent outdoors reduces the onset and progression of myopia	The cited literature indicated that time spent outside has a positive effect on the onset and progression of myopia. Increasing this time can be an easy way to reduce the risk of developing myopia and hold up the progression.
**53**	Donoghue, O., O'Connell, M. & Kenny, R.A. (2016). *Walking to Wellbeing: Physical Activity, Social Participation and Psychological Health in Irish adults aged 50 years and Older* . Dublin, Ireland: TILDA (The Irish Longitudinal Study on Ageing).	Walking to Wellbeing: Physical Activity, Social Participation and Psychological Health in Irish adults aged 50 years and Older.	E	IE	8172	50 and older	Irish population, 50 and older	Report that uses data collected during the first wave of The Irish Longitudinal Study on Ageing (TILDA)	walking and physical activity in general	1) better mental health status, better quality of life and overall wellbeing<br>2) higher participation in social activities and lower loneliness	Middle-aged and older adults walking 150 minutes per week report a better mental health status, better quality of life and overall wellbeing. Compared to those with low physical activity levels they are more socially active and have lower loneliness scores. Adults with low levels of physical activity are over twice as likely to have clinically relevant depressive symptoms.
**54**	Bratman, G.N., Hamilton, J.P., Hahn, K.S., Daily, G.C. & Gross, J.J. (2015). Nature experience reduces rumination and subgenual prefrontal cortex activation. *Proceedings of the National Academy of Science*, 112(28), pp.8567–8572.	Nature experience reduces rumination and subgenual prefrontal cortex activation.	B	US	38	40 to 59 years at first measure ments; 60 to 79 years at the follow-up testing	healthy individuals residing in urban environments	Comparison of brain activity during a 90 min walk in an urban or natural environment	walking	1) reduced risk for mental illness	A 90-minute walk in a natural setting, decreased both self-reported rumination and neural activity in an area of the brain linked to risk for mental illness. No such effect appeared in the urban walks and so the authors suggest that accessible natural areas within urban contexts may be a critical resource for mental health in a rapidly urbanizing world.

Ref. Number	Full Reference	Title in English	Meth. Code*	Country	Participants			Study Description	Type of Sport	Core Outcomes	Key Findings
					Number	Age	Population				
**55**	Brown, D. K., Barton, J. L., Pretty, J. & Gladwell, V. F. (2014). Walks4Work: Assessing the role of the natural environment in a workplace physical activity intervention. *Scandinavian Journal of Work, Environment & Health* , 40(4), pp.390-400.	Walks4Work: Assessing the role of the natural environment in a workplace physical activity intervention.	B	UK	73	adults	office workers	Study on the health benefits of an 8-week intervention of 20 minutes lunchtime walking conducted twice a week comparing different environments. Office workers have been randomly assigned to three groups: control, nature walking or walks in built environment.	walking,<br>green exercise	1) improved perceived mental health	3 groups of healthy indivduals participated in the trial - a nature walking, an urban walking and a control group (2 times per week). Self reported mental health improved for the nature walking group only. However, the hypothesis that repeated walks in nature would increase mental health could not be proven. The intensity of the physical intervention was evaluated as being insufficient for modifying cardiovascular health parameters and for supporting longer term adherence.
**56**	Weng, P. & Chiang, Y. (2014). Psychological Restoration through Indoor and Outdoor Leisure Activities. *Journal of Leisure Research,* 46(2), pp.203-217.	Psychological Restoration through Indoor and Outdoor Leisure Activities.	C	TW	203	18-26 years	Students	Exploring the effects of leisure activities on anxiety reduction and attention restoration, comparisons were made between the effect of participation in various indoor and outdoor leisure pursuits	walking, exercising + also gardening, internet surfing and chatting	1) improved mental health<br>2) restoring attention	Moderate leisure activity such as walking was the best outdoor activity for improving mental health. Chatting also reduced anxiety and restored attention. Surfing the internet and exercising both failed to significantly improve mental health. Outdoor activities were seen to be better for restoring attention compared to indoor activities.
**57**	Mitchell, R., (2013). Is physical activity in natural environments better for mental health than physical activity in other environments? *Social Science & Medicine,* 91, pp.130-134.	Is physical activity in natural environments better for mental health than physical activity in other environments?	E	UK	1890 (General Health Questionaire models) and 1860 (Warwick Edinburgh Mental health and Wellbeing Score models)	16 years and older	Scottish population over 15	Study on the synergistic effects between the psychological benefits of physical activity, and the restorative effects of contact with a natural environment using data from the Scottish Health Survey 2008, a large, real-world population.	green exercise in general	1) lower risk of poor mental health	Results suggest that physical activity in natural environments is associated with a reduction in the risk of poor mental health to a greater extent than physical activity in other environments, but also that activity in different types of environment may promote different kinds of positive psychological response. Each additional visit to a natural environment for physical activity per week could be associated with about a 6% lower risk of poor mental health. However, the authors could not proove a clear association between regular use of natural environments and greater overall wellbeing.
**58**	Duvall, J. & Kaplan, R. (2013). *Exploring the Benefits of Outdoor Experiences on Veterans* . San Francisco, California: Sierra Club Military Families and Veterans Initiative.	Exploring the Benefits of Outdoor Experiences on Veterans.	D	US	98	n/i	veterans	Research on the potential benefits of veterans' participation in multi-day group-based outdoor recreation experiences. Four organizations, offering 12 different programs each lasting 4-7 days, were included in the study.	group-based outdoor recreation activities such as backpacking and canoeing	1) benefits in psychological health and well-being in veterans<br>2) improved social functioning, life outlook and activity engagement<br>3) appealing therapeutic treatment for veterans	The findings suggest that group-based nature recreation can help veterans struggling with serious health problems. Results showed improved psychological well-being, social functioning, life outlook and activity engagement and also suggest a link between the activities and long-term psychological well-being.<br>The changes were particularly strong for veterans who had initially reported more severe ongoing health issues.
**59**	Mutz, M., & Müller, J. (2016). Mental health benefits of outdoor adventures: Results from two pilot studies. In *Journal of adolescents* , 49, pp.105-114.	Mental health benefits of outdoor adventures: Results from two pilot studies.	C	DE	12 and 15 (+7 controls)	over 14 years	youths and young adults	Evaluation of two pilot studies: the school project "Crossing the Alps", a nine-day hike through the German, Austrian, and Italian Alps; and the University project "Friluftsliv" eight days in the Norwegian wilderness	outdoor and adventure education programs namely one hiking and one Friluftsliv program	1) increase in life satisfaction<br>2) increase in mindfulness, happiness, self-efficacy<br>3) lower perceived stress	Participants of the hiking project reported an increase in life satisfaction, mindfulness and a decrease in perceived stress'; Friluftsliv participants scored higher than the control group in life satisfaction, happiness, mindfulness, and self-efficacy and lower in perceived stress.

Ref. Number	Full Reference	Title in English	Meth. Code*	Country	Participants			Study Description	Type of Sport	Core Outcomes	Key Findings
					Number	Age	Population				
**60**	Sandford, R., Duncombe, R. & Armour, K. (2008). The Role of Physical Activity/Sport in Tackling Youth Disaffection and Anti-Social Behaviour. *Educational Review* , 60(4), pp.419-435.	The Role of Physical Activity/Sport in Tackling Youth Disaffection and Anti-Social Behaviour.	C	UK	7000	pupils	disaffected youth and pupils showing anti-social behaviour	Evaluation of the HSBC/Outward Bound project and Youth Sport Trust/BSkyB "Living For Sport" programme	HSBC/Outward Bound programs include outdoor/ adventurous activity experiences; Youth Sport Trust/BSkyB include climbing, abseiling, horse-riding, skiing etc.	1) positive impact on youth development especially on behavior and attendance of disaffected or disadvantaged youths<br>2) re-engagement of disengaged or at risk young people<br>3) improved levels of self-esteem and personal confidence<br>4) increased happiness<br>5) improved interpersonal skills	Both projects had a positive impact on the behaviour and attendance rate. The results show that engagement in lessons and relationships with both teachers and peers improved and were sustained. Qualitative data from interviews with teachers highlight the following benefits: the majority of pupils appear happier, more engaged within lessons; less disruptive in large group situations and more able to work with others; they experience better relationships with both their peers and school staff and show improved levels of personal confidence. However, impacts were identified to be individualised and context-specific, with these being sustained when specific project crtieria were applied.
**61**	Marselle, M.R., Irvine, K.N. & Warber, S.L. (2014). Examining Group Walks in Nature and Multiple Aspects of Well-Being. *Ecopsychology,* 6(3), pp.134-147.	Examining Group Walks in Nature and Multiple Aspects of Well-Being.	D	UK	1991	over 18	adults	Evaluation of the Walking for Health program in England; to identify the mental, emotional, and social well-being benefits from participating in group walks in nature compared to a control group	group walks in nature	1) increased mental health with lower depression, perceived stress and negative affect, as well as enhanced positive affect and mental well-being	Results suggest that nature-based group walks lead to significantly lower depression, perceived stress, and negative affect, as well as enhanced positive affect and mental well-being. Additionally, the authors highlight that group walks in a natural setting seem to mitigate the effects of stressful life events on perceived stress and negative affect. The activities also synergise with physical activity to improve positive affect and mental well-being.
**62**	Sheng-Hshiung, T., Wei-Rong, L. & Tien-Ming, C. (2015). Toward a Structural Model of Challenge Experience in Adventure Recreation. *Journal of Leisure Research,* 47(3), pp.322-336.	Toward a Structural Model of Challenge Experience in Adventure Recreation.	E	TW	949	18 to 45 years	adventure recreationists	Study on the effects of adventure recreation on flow experience and psychological well-being; one time measurement after self chosen activity	scuba diving, high-altitude mountaineering, white water rafting	1) psychological well-being<br>2) flow experience<br>3) satisfaction	Adventure recreation and experiencing challenges showed a positive impact on flow experience and, by extension, on satisfaction, and psychological well-being.
**63**	Šuc, N., Lešnik, B. & Erpič, SC. (2015). Differences in self-concept among persons with disabilities due to practising adaptive alpine skiing. *Kinesiologia Slovenica* , 21(3), pp.34-42.	Differences in self-concept among persons with disabilities due to practising adaptive alpine skiing.	C	SI	unclear	39.8 ± 7.6 (IG); 36.7± 7.2 (CG)	recreational skiers with disabilities caused by injuries	Survey on the therapeutic effects of skiing on self-concept in persons suffering from an acute injury that led to a disability; carried out with two groups of adults comparing recreational alpine skiers with a control group	adaptive alpine skiing	1) rehabilitation, better self-concept and better self-perception of persons with disabilities after acute injuries	The authors recommend adaptive skiing as rehabilitation of self-concept for persons suffering from disabilities as a result of an acute injury. The results show that the skiers reported a positive self-concept despite their acquired injury. Compared to the control group, skiers showed significantly improved aspects of self concept: namely positive physical and personal self, identity, self-satisfaction, self-evaluation, are more adjusted and less neurotic.
**64**	Dorsch, T. E., Maxey, M. & Richards, A. R. (2016). The effect of an outdoor recreation program on individuals with disabilities and their family members: a case study. *Therapeutic Recreation Journal,* L (2), pp.155-171.	The effect of an outdoor recreation program on individuals with disabilities and their family members: a case study.	F	US	17	23 to 68 years	10 male, 7 female; (5 participants with various disabilities), 4 family members and 8 staff members.	Qualitative study (via semi structured interviews and focus groups) with participants, family members and staff at an outdoor recreation program for people with disabilities. Focus groups were conducted with all participants as well as in separate groups (participants, family members, staff)	various outdoor recreation activities - downhill skiing, kayaking, canoeing, snowshoeing, hiking, rock climbing, camping	1) increased confidence<br>2) increased personal skills<br>3) enhanced relationships<br>4) increased quality of life<br>5) sense of community<br>6) integration of disabled and overcoming barriers to participation in physical activity	The following themes came out of the focus groups. 1. Participants percieve social barriers to participation in physical activity (inhibited self esteem and confidence, percieved limitations, family as a barrier to participation) 2. Common ground provided ways to overcome these (Encourage a sense of community, see the person first, making the impossible possible) 3. participants percieved intra and interpersonal benefits (increased confidence and skill, enhanced relationships, elevated quality of life).
**65**	Ebner-Priemer, U., & Reichert, M. (2014). *Lauf Dich glücklich! Sport gegen depressive Verstimmungen. Untersuchung der Veränderung von Stimmung durch Bewegungs-/Sportinterventionen bei Studierenden mit Erkrankungen im depressiven Spektrum mittels ambulantem Assessment.* Karlsruhe: Karlsruher Institut für Technologie / Institut für Sport und Sportwissenschaft.	Run for happiness! Sport against depression. Evaluation of a sport intervention with students suffering from depression symptoms	D	DE	n/i	n/i	students with depression symptoms	10 weeks running intervention with a pre-post design, 45 min group training, 3 times a week. The design of the running training was acc. to NICE-guidelines recommendations and acc. to actual research results of studies on sport with people with depression symptoms (e.g. Stanton & Reaburn, 2013)	outdoor running	1) increased well-being<br>2) decreased depression<br>3) better mood<br>4) decreased rumination	The running intervention led to a significant decrease in depression symptoms as well as a significant increase in reported wellbeing in the pre-post comparison. Furthermore, results showed better mood and decreased rumination.

Ref. Number	Full Reference	Title in English	Meth. Code*	Country	Participants			Study Description	Type of Sport	Core Outcomes	Key Findings
					Number	Age	Population				
**66**	Federal, Provincial, and Territorial Governments of Canada. (2014). *2012 Canadian Nature Survey: Awareness, participation, and expenditures in nature-based recreation, conservation, and subsistence activities.* Ottawa, ON: Canadian Councils of Resource Ministers.	2012 Canadian Nature Survey: Awareness, participation, and expenditures in nature-based recreation, conservation, and subsistence activities.	E	CA	15000 + a selection of nearly 9,000 Web panelists	adults (age 18 and older)	Canadian adults	national survey on the awareness, participation, and expenditures in nature-based recreation	all nature-based recreation including hiking, climbing and horseback riding	1) contribution to quality of life	The survey illustrates that nature-based activities make a significant contribution to the national economy and individual Canadians' quality of life. For half of all Canadians, having access to nature is an important reason for their choice of residence.
**67**	Clough, P., Mackenzie, S.H., Mallabon, L., & Brymer, E. (2016). Adventurous physical activity environments: A mainstream intervention for mental health. In *Sports medicine* , 46(7), pp.963-968.	Adventurous physical activity environments: A mainstream intervention for mental health.	H	UK	n/a	n/a	n/a	Literature research that demonstrates why adventurous physical activity should be considered a mainstream intervention for positive mental health and well-being.	adventure activities	Enhanced psychological health and well-being through<br>1) increased positive affect, self-efficacy and resilience<br>2) opportunities to experience challenges, autonomy, competence and relatedness, nature connectedness and intense emotions<br>3) opportunities to fulfil basic psychological needs of autonomy, competence and relatedness<br>4) increased activity levels	Adventurous physical activities (APA) are linked to a range of psychological health and well-being outcomes that go beyond 'character building' concepts. As a conclusion of a literature review the authors present the following benefits: 1. APA increase positive psychological outcomes such as positive affect, self-efficacy and resilience, 2. APA provide opportunities to overcome challenges, to have optimal experiences and to experience intense emotions, 3. APA provide opportunities to fulfill basic psychological needs of autonomy, competence and relatedness, 4. APA facilitate feelings of connectedness to nature, 5. APA increase physical activity levels
**68**	Crust, L., Henderson, H., & Middleton, G. (2013). The acute effects of urban green and countryside walking on psychological health: A field-based study of green exercise. *International journal of sport psychology, 44* (2), pp. 160-177.	The acute effects of urban green and countryside walking on psychological health: A field-based study of green exercise.	C	UK	83	(M age = 62.91, s = 9.33)	recreational walkers	Comparison between the effects of walks in urban green space and walks in the countryside on markers of psychological health	walking	1) increases in positive affect and decreases in negative affect<br>2) increases in self-esteem<br>3) higher sport adherence due to higher enjoyment	Significant increases in positive affect and decreases in negative affect followed walk completion. Significant increases in self-esteem were found, with countryside walkers reporting significantly higher post-walk self-esteem than urban green walkers. Significantly higher levels of enjoyment were reported by countryside walkers which the authors argue is a determinant of greater sport adherence.
**69**	Hayhurst, J., Hunter, J., Kafka, S. & Boyes, M. (2015). Enhancing Resilience in Youth through a 10-Day Developmental Voyage. *Journal of Adventure Education and Outdoor Learning* , 15(1), pp.40-52.	Enhancing Resilience in Youth through a 10-Day Developmental Voyage	C	NZ	126 (1st study) plus 146 (2nd study)	mean age between 16.42 and 17.46 in different groups	youth	Two case studies on the effect of a 10 days sailing expedition on resilience in youth. Study 2 extended the pre-post-design by a follow-up test five months following the voyage.	voyage - sailing expedition	1) increased resilience<br>2) increased self-esteem, social effectiveness and self-efficacy	The results schowed an increased resilience over the course of the sailing expedition and in a five months follow up this was sustained. Significant increases could also be shown for self-esteem, social effectiveness and self-efficacy.
**70**	Barton, J., Griffin, M. & Pretty, J. (2011). Exercise, nature and socially interactive based initiatives improve mood and self-esteem in the clinical population. *Perspectives in Public Health,* 132(2), pp.89-96.	Exercise, nature and socially interactive based initiatives improve mood and self-esteem in the clinical population.	C	UK	53	21 to 83 years, with a mean age of 53.0 years (SD = 15.4)	clinical population with mental health problems	study that compares two existing group-based health promotion initiatives (a social club and a swimming group) to a new green exercise programme	green exercise (countryside and urban park walks)	1) improved self-esteem<br>2) improved mood<br>3) therapeutic treatment for people with mental health problems	The treatment led to an improvement in self-esteem and mood in people experiencing mental ill health. The change in self-esteem was significantly higher for the green exercise group compared to the social activities programme. Overall, green exercise as a health-promoting initiative was shown to be equally as effective as existing programmes. The authors therefore suggest a potential 'green' approach to mental healthcare and promotion by combining exercise, nature and social components. Initiatives like this could be useful means for therapeutic treatment for people with mental health problems.
**71**	Barton, J. & Pretty, J. (2010). What is the Best Dose of Nature and Green Exercise for Improving Mental Health? A Multi-Study Analysis. *Environmental Science & Technology.* 44(10), pp.3947–3955.	What is the Best Dose of Nature and Green Exercise for Improving Mental Health? A Multi-Study Analysis.	A	UK	1252	4 age groups included (under 30, 31-50, 51-70, over 70)	children, adults and elderly	Meta-analysis to assess the best regime of dose(s) of acute exposure to green exercise required to improve self-esteem and mood (indicators of mental health)	green exercise including cycling, walking, water sports and horse-riding	1) improved self-esteem<br>2) improved mood	The authors highlight positive returns from short engagements in green exercise and confirm that the environment provides an important health service. Over all of the studies the meta-analysis showed improvements in both self-esteem and mood for every green environment, however, the presence of water seems to generate greater effects. The results also show some differences in participating groups. For self esteem, the younger as well as mentally ill participants showed greatest improvements while for mood, the least change was in the younger and older participants.

Ref. Number	Full Reference	Title in English	Meth. Code*	Country	Participants			Study Description	Type of Sport	Core Outcomes	Key Findings
					Number	Age	Population				
**72**	Pretty, J., Peacock, J., Hine, R., Sellens, M., South, N. & Griffin, M. (2007). Green exercise in the UK countryside: Effects on health and psychological well-being, and implications for policy and planning. *Journal of Environmental Planning and Management,* 50(2), pp.211-231.	Green exercise in the UK countryside: Effects on health and psychological well-being, and implications for policy and planning.	D	UK	263	13 - 84 years; average was 47.8 ± 18.2 years	active and healthy people	Study on the effects of 10 green exercise case studies on mental health and wellbeing	green exercise including walking, cycling, horse-riding, fishing, canal-boating and conservation activities	1) improved self-esteem<br>2) decreased total mood disturbance	Results were similar for all 10 case studies. Self-esteem and mood were found not to be affected by the type, intensity or duration of the intervention. Green exercise led to a significant improvement in self-esteem and a decrease in total mood disturbance (with anger-hostility, confusion-bewilderment, depression-dejection and tension-anxiety all improving post-activity).
**73**	Hansmann, R., Hug, S.M. & Seeland, K. (2007). Restoration and stress relief through physical activities in forests and parks. *Urban Forestry & Urban Greening,* 6(4), pp.213-225.	Restoration and stress relief through physical activities in forests and parks.	D	CH	164	15 and older; 49.4% were over 50 years old	residents of Zurich, Switzerland	Field survey with an ad-hoc sample on restorative effects of visiting an urban forest and a city park in Zurich	outdoor recreation in general including jogging, biking or walking	1) restoration from stress and symptoms of headaches, increase in feeling well-balanced<br>2) outdoor recreation in cities as low cost possibilties for everyone	Outdoor recreation in parks or forests led to a significant improvement in symptoms of stress and headaches, and feeling of being well balanced increased significantly. Positive effects increased with length of visit, and individuals practicing sports showed significantly higher improvements than those engaged in less strenuous activities (e.g. relaxing).
**74**	Berman, Marc G., Kross, E., Krpan, K.M., Askren, M.K., Burson, A., Deldin, P.J., Kaplan, S., Sherdell, L., Gotlib, I.H. & Jonides, J. (2012). Interacting with nature improves cognition and affect for individuals with depression. *Journal of Affective Disorders,* 140(3), pp.300–305.	Interacting with nature improves cognition and affect for individuals with depression.	B	CA	20	mean age = 26	individuals with major depressive disorder (MDD)	Effects of a 50 minutes walk in natural compared to urban environments in individuals with major depressive disorder	walking in nature	1) cognitive and affective benefits, increase in memory span and mood<br>2) supplement to existing clinically treatments for individuals with major depressive disorder (MDD)	Results show cognitive and affective benefits of interacting with nature in individuals with major depressive disorder (MDD). The memory span increased significantly after the nature walk and mood also improved. However the mood effects showed no correlation with the memory effects, which presumes separate mechanisms. As a conclusion the authors suggest interacting with nature should be viewed as a useful supplement to existing clinically treatments.
**75**	Focht, B. C. (2009). Brief Walks in Outdoor Laboratory Environments. *Research Quarterly for Exercise and Sport,* 80(3), pp.611 -620.	Brief Walks in Outdoor Laboratory Environments.	C	US	35	mean age - 22.14 years	Active young women	A comparison of the effect of brief walks completed in outdoor and laboratory environments on affective responses, enjoyment, and intention to walk for exercise. 35 active women walked for 10minutes at a self selected intensity in outdoor and laboratory environments.	walking	1) increased affective states and enjoyment<br>2) higher intention for future participation	Both walks improved affective responses. However greater pleasant affective states, enjoyment and intention for future participation was shown with outdoor walking. The findings suggest that environment influences the affective responses to brief walks and show that affective states experiences during walking are related to theoretical determinants of physical activity.
**76**	Matsouka, O., Bebetsos, E., Trigonis, I. & Simakis, S. (2010). The effects of an outdoor exercise program on mood states among the elderly. *World Leisure Journal,* 52(1), pp.34-40.	The effects of an outdoor exercise program on mood states among the elderly.	C	GR	45 (30 exercise group, 15 control)	60 to 75 years	sedentary elderly people aged 60 to 75 (who had not been involved in exercise for at least 6 months prior to the exercise program)	Two groups were monitored (an exercise group n=30, and a control group n=15) with exercise was performed for approx 45 minutes, two times a week for 12 weeks by the exercise group. Exercise intensity was between 50-75% of Maximum heart rate. Control group did not participate in any form of exercise. Participants were pre- and post- tested for positive engagement, revitalization, tranquility and physical exhaustion.	Based on the long-term physical activity workshop, outdoor leisure activities, bodyweight exercises and callisthenic exercises took place in the natural outdoor environment	1) improved positive engagement and mood<br>2) revitalization<br>3) enhanced tranquility<br>4) reduced physical exhaustion	Exercise group had significantly more positive mood profiles than non-exercisers (control group). The improvements in positive engagement and mood, revitalization, enhanced tranquilityand reduced physical exhaustion directly corresponded to their participation in the programme.
**77**	Townsend, M. & Weerasuriya, R. (2010). *Beyond Blue to Green: The benefits of contact with nature for mental health and well-being* . Melbourne, Australia: Beyond Blue Limited.	Beyond Blue to Green: The benefits of contact with nature for mental health and well-being.	A	AU	n/a	n/a	n/a	literature review including current Australian and international research on the links between mental health and well-being and green spaces; focus lies on depression and anxiety	open green space activites including walking and hiking	1) mental health benefits such as improved mood, lower levels of anxiety, lower stress levels, lower levels of depression and increased physical activity	The authors present a vast range of evidence on the relationship between mental health and visits to nature, which includes improved mood, lower levels of anxiety, lower stress levels, lower levels of depression and increased physical activity. Furthermore, they give a summary on social and mental health benefits of nature-based activities in children and on psychological benefits of a perceived green neighborhood and proximity to green space.

Ref. Number	Full Reference	Title in English	Meth. Code*	Country	Participants			Study Description	Type of Sport	Core Outcomes	Key Findings
					Number	Age	Population				
78	Bodin, M., & Hartig, T. (2003). Does the outdoor environment matter for psychological restoration gained through running? *Psychology of Sport and Exercise,* 4(2), pp.141-153.	Does the outdoor environment matter for psychological restoration gained through running?	B	CA	12	26 to 46 years, mean age: 39.7 years	adult runners with over 3 years running experience	Field experiment to analyse the moderating effect of environment (park/nature reserve vs. urban setting) on attentional and emotional restoration followed by a 1 hour run (maximum length of 14 kilometers with relaxed pace)	running	1) decline in anxiety/depression and anger<br>2) green enviroment perceived as more restorative	The running intervention led to changes such as a significant decline in anxiety/depression and anger. Participants preferred the park over the urban environment and perceived it as more psychologically restorative. However, there was no significant difference between the two environmental settings (with a very small sample size and medium sized effects an tranquility and anxiety/depression).
79	Krenichyn, K. (2006). 'The only place to go and be in the city': Women talk about exercise, being outdoors, and the meanings of a large urban park. *Health & Place,* 12(4), pp.631-643.	'The only place to go and be in the city': Women talk about exercise, being outdoors, and the meanings of a large urban park.	F	US	n/i	n/i	women	Qualitative study on women's perceptions of physical activities in an urban park in Brooklyn, NY	outdoor recreation in general including activities like walking, running, and cycling	1) feelings such as pleasure and enjoyment, meditation, release of stress and independence<br>2) park as a social place for interaction<br>3) engaging social groups in physical activity that are likely to not fulfill recommended activity levels<br>4) greater enjoyment of physical activity	Interviewed women described their experiences of park visits with feelings such as pleasure and enjoyment, meditation and release of stress. The study also underlines the importance of nearby or everyday outdoor environments for encourageing physical activity and of parks as a place for social interaction.
80	Bratman, G.N., Daily, G.C., Levy, B.J. & Gross, J.J. (2015). The benefits of nature experience: Improved affect and cognition. *Landscape and Urban Planning,* 138, pp.41–50.	The benefits of nature experience: Improved affect and cognition.	B	US	60	adults	adults	Study comparing the effects of walks in natural and urban environment on affect and cognition	walking	1) improvements in affect (e.g., decrease in anxiety and rumination) and 2). cognition (complex working memory span task)	The results support the idea that green exercise can improve affect and cognitive function. Nature experience during exercise produced clear benefits for affect (i.e., decrease in anxiety and rumination) and some benefitial effects for cognitive function (complex working memory span task).
81	Levin, B.J. & Taylor, J. (2011). Depression, Anxiety, and Coping in Surfers. *Journal of Clinical Sport Psychology* 5(2), pp.148-165.	Depression, Anxiety, and Coping in Surfers.	E	US	100	n/i	surfers	Cross-sectional study; one time of measurement and comparison to prevalence data from literature	surfing	1) mental health benefits: reduced incidence of depression and anxiety<br>2) better coping strategies in stressful situations	As a hybrid of meditative and athletic experience surfing is linked to several mental health benefits. Compared to the general populace, interviewed surfers reported significantly fewer symptoms of depression and anxiety, and employed emotion-based coping responses to stressful situations significantly less. Results also showed that surfers employed avoidance-based coping strategies more frequently than the general populace
82	Stanton, R. & Reaburn, P. (2014). Exercise and the treatment of depression: A review of the exercise program variables. *Journal of Science and Medicine in Sport.,* 17(2), pp.177-182.	Exercise and the treatment of depression: A review of the exercise program variables.	A	AU	n/a	18 to 65 years	n/a	Systematic review on exercise and depression including five RCTs published since 2007. Most programs were performed three times weekly and of moderate intensity.	aerobic exercise, either treadmill or outdoor walking, stationary cycle or elliptical cross trainer exercise	1) reduced depression	There is evidence for the use of supervised aerobic exercise, undertaken 3-4 times weekly at moderate intensity for a minimum of nine weeks in the treatment of depression.
83	Mackay. G. J. (2010). The effect of "green exercise" on state anxiety and the role of exercise duration, intensity and greenness: A quasi-experimental study. *Psychology of Sport and Exercise,* 11, pp.238-245.	The effect of "green exercise" on state anxiety and the role of exercise duration, intensity and greenness: A quasi-experimental study.	D	AU	101	mean age - 43.83, males between 23-82, females between 20-66.	participants were from pre-existing outdoor exercise groups	A quasi-experimental study investigating the impact of green exercise on levels of anxiety involving eight pre-existing outdoor exercise groups. Pre and post- test questionnaires were completed collecting demographics and a State-trait anxiety inventory for adults (STAI) score. Participants rated exercise intensity using the borg scale.	Road cycling, mountain running, orienteering, cross-country running, boxercise, mountain biking, kayaking, walking	1) decreased anxiety	There was a significant reduction in participants' anxiety state following green exercise experiences. However there was a significant interaction between anxiety changes and the type of green exercise. The largest anxiety reductions were reported by road cycling, boxercise and mountain biking groups. Exercise intensity and duration did not impact on anxiety state changes, however higher degrees of perceived environmental greenness were associated with larger reductions in anxiety.
84	Sand, M. S. (2015). *Die Auswirkungen des sechsmonatigen Segel-Schulprojektes Klassenzimmer unter Segeln auf die Persönlichkeitsentwicklung Jugendlicher.* Hamburg: Czwalina.	The effects of a six month sailing school project on personality development in youth	C	DE	30 intervention + 30 control group	mean age: 15,13 years and 15,18 control	children of different grammar schools	Explorative pilot study on changes of personality and learning through a 6 month sailing intervention called "class room under canvas"	sailing	1) decrease in social anxiety<br>2) increase in emotional passive coping<br>3) better perception of meaningful living vs. depression	The sailing programme showed several positive effects on personality development: a decrease in social anxiety, an increase in emotional passive coping, reduced feelings of performance pressure and a better perception of "meaningful living vs. depression" in a specific personality scale

Ref. Number	Full Reference	Title in English	Meth. Code*	Country	Participants			Study Description	Type of Sport	Core Outcomes	Key Findings
					Number	Age	Population				
85	Marques, A. P. (2010). Protocolo ARC aplicado en la Escola de la Foresta. *Anduli: revista andaluza de ciencias sociales,* (9), pp.149-163.	ARC Protocol applied to La Floresta School.	F	ES	n/a	n/a	adults having drug problems or other social exclusion risks	Description of a program applied to the rehabilitation of drug addicts by the use of outdoor activities and experiences of controlled risk	outdoor sports in general / risk under control	1) vital strength and a higher will to live<br>2) enhanced feeling of their body, discovering the pleasure of achievement<br>3) habitus of team work and group development for motivation and self security<br>4) rehabilitation of drug addicts<br>5) improved behaviour and habitus of adults having drug problems or other social exclusion risks<br>6) nature as an environment for an intense contact with one's self	The designed programme, based on outdoor activities and experiences of controlled risk, improve the behaviour and habitus of adults who have drug problems or other social exclusion risks. Participants experience three phases: 1) an initial shock, 2) learning to control their fear and discovering the pleasure of achievement and 3) discovery of other pleasures including nature.
86	Dimmock, K. (2009). Finding comfort in adventure: experiences of recreational<br>SCUBA divers. *Leisure Studies*, 28(3), pp.279-295.	Finding comfort in adventure: experiences of recreational SCUBA divers	F	AU	27	20 to 60 years	All participants were certified in open water dives (range of ability)	A study of participants' comfort during an adventure-based experience (SCUBA diving). Interviews were held with certified open water divers with a variation in experience level and involvement with the activity	scuba diving	1) physical, social, psychological and visual experiences of comfort	The interpretation of interiew data revealed four contexts of comfort that emerged: physical, social, psychological and visual.
87	Vălev, Y. & Dimitrov, V. (2015). Въздействие на конния спорт върху човека. *Sport & nauka* , 59 (5), pp. 28-36.	Influence of the equestrian sport on humans.	H	BG	n/a	n/a	n/a	This literature review reflects on the positive effects of equestrian sports.	horseriding	1) euphoria, relaxation and positive results through contact with horses<br>2) hippotherapy	The equestrian sport is a fast growing activity and is gaining greater popularity. Participants encounter euphoria, relaxation and other positive results from the contact with horses. These positive effects can be used in therapeutic aspects of hippotherapy.
88	Leithäuser, R., & Beneke, R. (2013). Sport bei ADHS – Plan für Desaster oder verschenkte Ressource? In *Deutsche Zeitschrift für Sportmedizin, 64* (10), pp.287-292.	Sports for ADHD-patients – plan for disaster or wasted resource?	H	DE	n/a	n/a	healthy populations and ADHS patients	No intervention, theoretical text that argues for a general positive effect of physical activity (especially those performed in natural settings) on behaviour and cognition in ADHS patients.	outdoor sport in general	1) positive effects on behaviour and cognition<br>2) improves attention and power to concentrate, better cognitive performance<br>3) improve of self-esteem, self-confidence and social skills<br>4) therapeutic low-cost, non-drug treatment for ADHD patients	Evidence for positive effects on behaviour and cognition is shown for healthy populations as well as for ADHD sufferers. Physical activity results in better attention and concentrativeness, better cognitive performance and better social behavior as it enables ADHD-sufferers to experience and accept own limits and those of others, and to learn and practice fair play. Positive experiences linked to physical activity can improve self-esteem, self-confidence and social skills, which can also have a positive impact on other areas of life. The effect of decreased ADHD syndroms was higher and longer-lasting/ more sustainable for activities in a natural outdoor setting both for group and single activities. Therefore, the authors see active programs in a natural environment as a possible low-cost, non-drugs treatment for ADHD patients.
89	Paquette, L., Brassard, A., Guérin, A., Fortin-Chevalier, J. & Tanguay-Beaudoin, L. (2014). Effects of a Developmental Adventure on the Self-Esteem of College Students. *Journal of Experiential Education* , 37(3), pp.216-231.	Effects of a Developmental Adventure on the Self Esteem of College Students.	C	CA	84	14 to 22 years	college students	Quasi-experimental study on the effects of a 5 months outdoor adventure program, compared to sports-only and travel only interventions	outdoor adventure	1) increase in self-esteem	The experimental group (outdoor adventure) showed positive and significant increases for both the athletic dimension of self-esteem and for global self-esteem (which is the general value that a person places on<br>themselves) after the intervention and two months later. This was higher than the sport or travel only groups.
90	Capurso, M. & Borsci, S. (2013). Effects of a Tall Ship Sail Training Experience on Adolescents' Self-Concept. *International Journal of Educational Research* , 58, pp.15-24.	Effects of a Tall Ship Sail Training Experience on Adolescents' Self-Concept.	D	IT	147	mean age of 13.18 years	adolescents with either a chronic diseases or some kind of physical or cognitive disability or youths at risk (from at-risk communities, school drop-outs or youngsters with a penal sanction)	Quasi-experimental study on the impact of a sail training education programme on the self-concept of adolescents having either a chronic diseases or some kind of physical or cognitive disability or youths at risk	sailing	1) positive, short-term effects on self-concept	The sailing intervention was for young people with a chronic disease or some kind of physical or cognitive disability or who were at risk. It led to significant improvememnts in competence and social elements within a standardised self-concept scale, directly after the experience but these were not sustained when the participants returned to their daily life contexts.

Ref. Number	Full Reference	Title in English	Meth. Code*	Country	Participants			Study Description	Type of Sport	Core Outcomes	Key Findings
					Number	Age	Population				
**91**	Vives Vilarroig, J. & Ruiz Bernardo, M. P. (2017). Programa asistido con caballos para la atención psicopedagógica de un adolescente con tda-h. *Quaderns digitals: Revista de Nuevas Tecnologías y Sociedad,* 84, pp.142-155.	Horse-assisted psychopedagogical programme for the intervention on an ADHD teenager.	F	ES	1	teenager	ADHD student	Case study evaluating the effects of a psycho-pedagogical intervention with horses in a student with ADHD.	horseriding	1) improved self-esteem and coping with problems in teenagers with ADHD<br>2) awareness of the reality and the impact of one's behavior on the social environment<br>3) improved self-regulation and social relations<br>4) treatment for ADHD students<br>5) high degree of motivation	The psychopedagogical intervention with horses has served to improve the self-esteem and coping with problems for a teenager with ADHD. The activity was valued as highly motivating and, from a qualitative results point of view, resulted in an increased awareness of his reality and the impact of behaviour on the social environment, improved self-regulation and social relations, which in turn increased self-esteem.
**92**	Burke, S. M. & Utley, A. (2013). Climbing towards recovery: investigating physically injured combat veterans' psychosocial response to scaling Mt. Kilimanjaro. *Disability & Rehabilitation,* 35(9), pp.732-739.	Climbing towards recovery: investigating physically injured combat veterans' psychosocial response to scaling Mt. Kilimanjaro.	F	UK	4	22 to 44 years	male veterans	Ethnographic study to explore the psychosocial effects of a 9 days climb of Mt. Kilimanjaro in veterans that have been wounded physically as a result of active duty in Afghanistan	trekking / mountaineering	1) psychosocial resource for recovery of serious injury<br>2) positive effects in self-determination, active coping and social support in veterans	Challenging activities may function as a psychosocial resource for recovery from serious injury. The findings show positive effects of self-determination, active coping and social support in participating veterans.
**93**	Clapham, E. D., Armitano, C. N., Lamont, L. S. & Audette, J. G. (2014). The Ocean as a Unique Therapeutic Environment: Developing a Surfing Program. *JOPERD: The Journal of Physical Education,* Recreation & Dance, 85(4), pp.8-14.	The Ocean as a Unique Therapeutic Environment: Developing a Surfing Program.	F	AU	17	5 to 17 years	Children with disabilities	Evaluation of an educational surfing program for children with disabilities; programme took place twice a weeke,with a duration of eight-weeks	surfing	1) benefits in social development and self-confidence for children with disabilities	The surfing programme for children with disabilities had many positive outcomes, such as gains in social development and self-confidence. Effects included the development and enhancement of children's strength, flexibility, range of motion, coordination, balance, and psychosocial development.
**94**	Bernardo, R. & Matos, M. (2003). Desporto aventura e auto-estima nos adolescentes, em meio escolar. *Revista Portuguesa de Ciências do Desporto,* 3 (1), pp.33–46.	Adventure Sports and self esteem on teenagers in school enviroment.	C	PT	66	teenagers	students from the 6º;7º;8º;9º and 10º grade	Evaluation on the psychological effects of an outdoor adventure activities programme on the self-esteem and physical self-perceptions of Portuguese school adolescents. The intervention had a duration of 10 weeks, with a total of 12 sessions and pre-post measurements.	adventure sports program: mountain bike, canoeing; climbing;, orienteering, hiking, camping; archery, rope activities	1) increased global and physical self-esteem	The authors suggest that there are some beneficial effects gained from the practice of adventure sports. The results showed some increases in global and physical self-esteem following the intervention. The authors noted more realistic levels of self-perception.
**95**	Palmberg, E. & Kuru, J. (2010). Outdoor Activities as a Basis for Environmental Responsibility. *The Journal of Environmental Education,* 31(4), pp.32-36.	Outdoor Activities as a Basis for Environmental Responsibility.	F	FI	36	11 and 12 years old	pupils	Qualitative study on the effects of environmental education on the development of pupils' affective relationship to the natural environment, their environmental sensitivity, and outdoor behavior, as well as their social relationships. Additionally, groups with differing experience levels have been compared.	canoeing, hiking, sailing, tent overnights, and camp schools	1) increase in self-confidence<br>2) enhanced empathic relationship towards nature<br>3) better social behavior and higher moral judgements<br>4) outdoor sports as a tool for environmental education<br>5) willingness to participate in future outdoor activities	Nature experiences contributed to the development of the pupils' self-confidence and feelings of safety, and their willingness to participate in future outdoor activities. Nature was valued as important by most of the pupils. However, those participants who were more experienced in outdoor activities seemed to have a strong and clearly definable empathic relationship to nature and also exhibited better social behaviour and higher moral judgements.
**96**	Mazzoni, E. R., Purves, P. L. & Southward, J. (2009). Effect of Indoor Wall Climbing on Self-Efficacy and Self-Perceptions of Children With Special Needs. *Adapted Physical Activity Quarterly,* 26, pp.259-273.	Effect of Indoor Wall Climbing on Self-Efficacy and Self-Perceptions of Children With Special Needs.	B	CA	46	6 to 12 years	Children with special needs (recieving services from a physio-therapist at school), Children had no experience in wall climbing and scored at or below the 15th percentile on the movement assessment battery for children.	Study on the effects of a 6 weeks indoor wall climbing program on perceptions of self in children with special needs. Participants were randomly assigned to the intervention or control group. Baseline assessments were taken 1 week prior to the sessions and a self efficacy questionnaire was completed after the first and sixth session.	indoor wall climbing	1) increased self-efficacy	Children's self efficacy and belayers ratings of children's efficacy improved significantly. The children's judgements of their athletic and social competence and global self-worth, however did not change over time or differ from the control group.

Ref. Number	Full Reference	Title in English	Meth. Code*	Country	Participants			Study Description	Type of Sport	Core Outcomes	Key Findings
					Number	Age	Population				
**97**	Moxham, L., Liersch-Sumskis, S., Taylor, E., Patterson, C. & Brighton, R. (2015). Preliminary Outcomes of a Pilot Therapeutic Recreation Camp for People with a Mental Illness. *Therapeutic Recreation Journal,* 49(1), pp.61-75.	Preliminary Outcomes of a Pilot Therapeutic Recreation Camp for People with a Mental Illness.	F	AU	27	21-71 years	n/a	5 day therapeutic recreation camp in the Australian bush for people with a shared experience of mental health	high ropes, rock climbing, tai chi, dance	1) increased confidence<br>2) increased social connectedness<br>3) enhanced individual responsibility<br>4) therapeutic tool for people suffering from mental illness	Participants perceived their confidence to have increased, their social connectedness to have grown and individual responsibility was enhanced. Due to the small sample size and methodology the positive findings cannot be widely generalised.
**98**	Panajotov, N. & Ajkora, E. (2012). Влиянието на катеренето върху нивата на самоактуализацията и емоционалната стабилност при юноши със заседнал начин на живот. *Sport & nauka* , 56(6), pp.123-131.	The impact of climbing on the levels of self-actualization and emotional stability in adolescents with a sedentary way of life.	C	TR	97	12 to 17 years	adolescents with a sedentary lifestyle	Case control study of a climbing intervention with adolescents leading a sedentary lifestyle. The program was run for 8-weeks with 4 days climbing per week. The effects were compared to a control group that did not participate in any activity.	indoor wall climbing	1) positive impact on self-actualization<br>2) positive impact on emotional stability	Climbing activities have a positive impact on self actualization and emotional stability of adolescents with a sedentary lifestyle.
**99**	Barton, J., Bragg, R., Pretty, J., Roberts, J. & Wood, C. (2016). The Wilderness Expedition: An effective life course intervention to improve young peoples well-being and connectedness to nature. *Journal of Experiential Education,* 39(1), pp.59-72.	The Wilderness Expedition: An effective life course intervention to improve young peoples well-being and connectedness to nature.	D	UK	130	11 to 18 years	adolescents	Effects of sixteen wilderness interventions in South Africa or in Scotland (5-11 days) on self-esteem and connectedness to nature in adolescent.	wilderness expeditions including activities like hiking, wild swimming, wild nature watching and canoeing	1) increased self-esteem<br>2) increased connectedness to nature	Wilderness interventions significantly increased self-esteem and connectedness to nature in adolescents. Regarding gender differences, males had a higher level of self-esteem in the pre-intervention measurement, but females levels increased more during the intervention. Relevant literature shows that connectedness to nature is associated with elements of psychological health and wellbeing, increased happiness and ecological behaviour. Low self-esteem is seen as a mental health risk factor, which is especially prevalent in females.
**100**	Lang, S. (2005). *Umweltthemen in der Sportpädagogik: Evaluation eines Schulsportprojektes als Beitrag zur Umweltbildung und Bildung für nachhaltige Entwicklung* . Augsburg: Universität Augsburg / Philosophisch-Sozialwissenschaftliche Fakultät / Institut für Sportwissenschaft.	The environmental issue and sport pedagogy. Evaluation of a project at secondary schools as a contribution to environmental education and education of sustainable development	C	DE	142	11 to 13 years	school children of different school types	bicycle riding intervention carried out on the way to and from a school camp with additional environmental education contents during the stay; pre-post evaluation on environmental consciousness, knowlegde, intended and effective actions directly after and half a year after the intervention	cycling	1) environmental awareness and behavior<br>2) opportunity to strengthen self-confidence	The cycling intervention led to an increase in knowledge of sustainability issues which sustained until the re-test. Furthermore the results show an increasing sensibility concerning environmental problems and an increase in environmentally friendly behavior. The author also states that cycling can be seen as a possibility to strengthen the self-confidence of children.
**101**	Glover, T., Chapeskie, A., Mock, S., Mannel, R. & Feldberg, H. (2011). *The Canadian Summer Camp Research Project.* University of Waterloo.	The Canadian Summer Camp Research Project.	F	CA	65 camp directors ; 1,295 campers	3-18 years; majority of participating campers (77%) were 7-14 years old	participants of youth summer camps	explorative study on the benefits of summer camps in Canada; evidence arises from camp directors' interviews and a survey with camp participants	outdoor sports in camps	1) social integration and citizenship<br>2) environmental awareness<br>3) self-confidence and personal development<br>4) emotional intelligence<br>5) attitudes towards physical activity	Direct interviews with Canadian camp directors revealed five themes of benefits they witnessed in their campers: social integration and citizenship, environmental awareness, self-confidence and personal development, emotional intelligence, and attitudes towards physical activity. While results showed a significant positive growth in all five areas for all groups, female campers, older campers and repeat participants tended to score higher on the observation instrument
**102**	Wheaton, B. (2017). Surfing through the life-course: silver surfers' negotiation of ageing. *Annals of Leisure Research,* 20(1), pp.96-116.	Surfing through the life-course: silver surfers' negotiation of ageing.	F	UK	11 (4 female, 7 male)	45 to 70 years	Recreational surfers	Qualitative study on the meanings that recreational surfing plays in participants' lives and identities as they grow older. In depth interviews were conducted with 11 surfers of different abilites along with participant observation providing insight into the different ways of understanding ageing.	surfing	1) active-ageing	Insight into how older surfers challenge discourses about ageing, physical activity and embodiment with implications for active ageing policy agendas.

Ref. Number	Full Reference	Title in English	Meth. Code*	Country	Participants			Study Description	Type of Sport	Core Outcomes	Key Findings
					Number	Age	Population				
**103**	Calsius, J., Courtois, I., Feys, P., Van Asch, P., De Bie, J. & D'hooghe, M. (2015). "How to conquer a mountain with multiple sclerosis". How a climbing expedition to Machu Picchu affects the way people with multiple sclerosis experience their body and identity: a phenomenological analysis. *Disability & Rehabilitation,* 37(26), pp.2393-2399.	"How to conquer a mountain with multiple sclerosis". How a climbing expedition to Machu Picchu affects the way people with multiple sclerosis experience their body and identity: a phenomenological analysis.	F	BE	9	n/i	n/i	Phenomenological analysis of the effects of a 5-day trekking to Machu Picchu on changes in identity and body awareness in people with multiple sklerosis;<br>A physical training schedule for several months prepared the participants before the trip.	trekking / mountaineering	1) positive effects on body awareness and identity in MS patients<br>2) therapeutical approach for people with MS	The MS patients who participated in the Machu Picchu trekking completed a unique kind of expedition outside their normal comfort zone. It had a deep and profound effect on body awareness and identity. It helped them to redevelop a harmonious relation between body, mind and the surrounding world, and helped to overcome the perceived disruption of their previous life by the illness.
**104**	Faber Taylor, A. & Kuo, F.E. (2008). Children With Attention Deficits Concentrate Better After Walk in the Park. *Journal of Attention Disorders,* 12(5), pp.402-409.	Children With Attention Deficits Concentrate Better After Walk in the Park.	B	US	17	7 to 12 years	children with ADHD	Effects of different environments of 20 min. walks in children with ADHD;<br>environments: a city park and two other well-kept urban settings	walking	1) therapeutic treatment for children suffering from ADHD<br>2) better attention performance	Children with ADHD concentrated significantly better after a walk in the park compared to a downtown or a neighborhood walk and achieved results that are comparable with medication by methylphenidate (commonly branded as Ritalin). The authors come to a conclusion that walking in natural settings can enhance attention not only in the general population but also in ADHD populations.
**105**	Kuo, F. E. & Taylor, A. F. (2004). A potential Natural treatment for Attenion-deficit/hyperactivity disorder: evidence from a national study. *American Journal of Public Health,* 94(9), pp.1580-1586.	A potential Natural treatment for Attenion-deficit/hyperactivity disorder: evidence from a national study.	E	US	452	Children were 5-18 years, parents did the survey about the children.	participants were parents or guardians of children with diagnosed ADHD	Examination of the impact of relatively "green" or natural settings on attention-deficit/hyperactivity disorder (ADHD) predominently measured through online survey data from parents whose children have been diagnosed with ADHD. Distinction and comparisions were made between activities in a 'green outdoor setting' or a 'built outdoor setting'	range of outdoor and indoor afterschool acitivites	1) reduced ADHD symptoms	Green outdoor settings reduced symptoms of ADHD in children more than activities conducted in other settings. Findings were consistent across age, gender, and income groups; community types; geographic regions; and diagnoses age, gender, and income groups; community types; geographic regions; and diagnoses.
**106**	Matos, M.G., Santos, A., Fauvelet, C., Marta, F., Evangelista, E.S., Ferreira, J. et al. (2017). Surfing for Social Integration: Mental Health and Well-Being promotion through Surf Therapy among Institutionalized Young People. *J Community Med Public Health Care,* 4(26), n.pp.	Surfing for Social Integration: Mental Health and Well-Being promotion through Surf Therapy among Institutionalized Young People.	D	PT	48	10 to 16 years	children and youth in foster care institutions	surf therapy intervention for children and youth in foster care institutions	surfing	1) intra- and interpersonal, social and emotional development<br>2) inclusion and therapy for at-risk young people<br>3) healthy lifestyle<br>promotion<br>4) improved effort and perseverance, problem-solving, time management<br>5) decrease in behavioural problems and in the total scale of difficulties	Results suggest that surf therapy had a number of positive intra- and interpersonal effects in vulnerable, at-risk young people. These include improved effort and perseverance, problem-solving, time management, social competencies, interpersonal relationships and emotional regulation. The tutors reported a statistically significant decrease in the emotional symptoms, behavioural problems and in a scale that assessed difficulties.
**107**	Henstock, M., Barker, K. & Knijnik, J. (2013). 2, 6, Heave! Sail Training's Influence on the Development of Self-Concept and Social Networks and Their Impact on Engagement with Learning and Education. A Pilot Study. *Australian Journal of Outdoor Education* , 17(1), pp.32-46.	2, 6, Heave! Sail Training's Influence on the Development of Self-Concept and Social Networks and Their Impact on Engagement with Learning and Education. A Pilot Study.	F	AU	5	students	disengaged youths	Pilot study on the impact of a sailing programme on the self-concept and social networking skills of at risk/disengaged youths and how this may influence students' engagement with learning and education.	sailing	1) increase in sense of purpose for learning and motivation to study<br>2) positive effects on development of social relationships and social networking skills<br>3) positive effects on general self-concept<br>4) re-engaging disengaged or at risk young people	Participation in the sailing program had a positive effect on development of social relationships and networking skills, general self-concept, motivation to study, and sense of purpose for learning.
**108**	Kudlacek, M., Bocarro, J., Jirasek, I. & Hanus, R. (2009). The Czech Way of Inclusion through an Experiential Education Framework. *Journal of Experiential Education* , 32(1), pp.14-27.	The Czech Way of Inclusion through an Experiential Education Framework	I	CZ	n/a	n/a	disabled	Theoretical statement including a case study of the Czech Outward Bound and reflections on several inclusive experiential education programs	outdoor education	1) inclusion of people with disabilities<br>2) personal development	The authors see outdoor education as one of the most appropriate settings for inclusive courses as participants learn experientially about differences. Away from mainstream society and environments, participants get opportunities for the development of social, physical, mental and spiritual aspects of themselves regardless of their abilities.

Ref. Number	Full Reference	Title in English	Meth. Code*	Country	Participants			Study Description	Type of Sport	Core Outcomes	Key Findings
					Number	Age	Population				
**109**	Caballero, P. J. (2015). Diseño, implementación y evaluación de un programa de actividades en la naturaleza para promover la responsabilidad personal y social en alumnos de formación profesional. *Cuadernos de Psicología del Deporte,* 15(2), pp.179-194.	Design, implementation and assessment of an outdoor activities programme to promote personal and social responsibility in vocational training students.	C	ES	43 (21 experimental group / 22 control group)	16 to 23 years	students	Effects of an outdoor activities intervention (5 months, 15 hours per week) on personal and social responsibility in students. The programme is based on the Hellison responsibility model and on Pedagogy of Adventure.	hiking, mountain biking, horseriding	1) positive effects on personal and social responsibility	The experimental group of students gained more positive effects of personal and social responsibility than those of the control group. This was also perceived by and verified for the teachers who had anecdotally observed this.
**110**	Beaumont, E. & Brown, D. H. K. (2015). 'Once a Local Surfer, Always a Local Surfer': Local Surfing Careers in a Southwest English Village. *Leisure Sciences,* 37(1), pp.68-86.	'Once a Local Surfer, Always a Local Surfer': Local Surfing Careers in a Southwest English Village.	F	UK	29	n/i	surfers or "exsurfers"	Ethnographic study using interviews with local surfers in a village with a significant population of local surfers to identify pathways and meanings of the sport practice. No intervention	surfing	1) positive cultural role, both for individuals and for the construction and maintenance of local community life	The study identifies shared experiential stages of local surfer careers that have been analysed in a community setting.<br>Findings highlight that shared experiences of a local surfing career play a positive cultural role, for individuals and for the construction and maintenance of local community life.
**111**	Hansen, K. & Parker, M. (2009). Rock Climbing: An experience with responsibility. *Journal of Physical Education, Recreation and Dance,* 80(2), pp.17-55.	Rock Climbing: An experience with responsibility.	F	US	not given	boys and girls aged 11 to 14 years	participants had not been successful in a school setting	Descriptive, qualitative study on a pedacogical climbing program. Participants took part in 75 minutes program on a university climbing wall once a week. At the end of each session they wrote individual reflections.	indoor wall climbing	1) pedagogical programme (character development curriculum) to teach responsibility, respect, teamwork, and trust<br>2) Self-development, self-motivation, contributing to the wellbeing of others	This article offers insight and guidance relating to the delivery of youth development programmes in climbing. It considers programme design to teach responsibility, respect, teamwork and trust but does not analyse the power of climbing to deliver these outcomes.
**112**	Widmer, M. A., Duerden, M. D. & Taniguchi, S. T. (2014). Increasing and Generalizing Self-Efficacy. The effects of adventure recreation on the academic efficacy of early adolescents. *Journal of Leisure Research,* 46(2), pp.165-183.	Increasing and Generalizing Self-Efficacy. The effects of adventure recreation on the academic efficacy of early adolescents.	C	US	262	Average of 13.6 years	Students	Quasi-experimental study focused on adolescents, including the use of treatment and comparison (control) groups. The intervention was a 2 week theory-based residential programme incorporating a range of outdoor activities delivered in 2004-2007. The purpose was to examine the effectiveness of using adventure recreation to increase outdoor recreation efficacy and generalize it to academic efficacy.	rafting, backpacking and exploration as part of an adventure programme	1) increased academic efficacy, attitudes, and motivations<br>2) increased outdoor self-efficacy	The participant group experienced a significant pre- to post-test increase in outdoor and academic measures. Adventure recreation programming may be an effective approach to facilitate the development of positive outcomes with applicability for both out-of-school and academic contexts.
**113**	Gešev, P. & Papazoglu, J. (2014). Проучване на взаимовръзките между изследвани показатели при неспортуващи ученици и ученици, занимаващи се допълнително с ветроходен спорт. *Sport & nauka* , 58(2), pp.36-43.	Research of the relationship between the investigated parameters of non-sportive students and students practicing sailing.	C	GR	121	14 to 16 years	students	This case control study investigates the development of motor skills in pupils through additional sailing lessons. Controls (n=77) took part in the regular PE classes, whereas the experimental group (n=44) got additional sailing classes.	sailing	1) improved motor skills	The study suggests that additional sailing activities have a positive impact on the development of improved motor skills in students.
**114**	Petkova, I. (2012). Ролята на спортните игри с елементи на ориентиране за укрепване на физическото и психическото развитие на деца от специализирана институция. *Sport & nauka* , 56(6), pp.96-106.	The role of sport games with emphasis to physical and psychological development of kids at special public institutions.	F	BG	28	6 to 7 years	children	Evaluation of the "Nature is my friend" project. Children take part in outdoor activities with a game character like orienteering and hiking.	outdoor activities with a game character like orienteering and hiking	1) increase of motor skills<br>2) positive effects on volitional qualities, endeavour and readiness to face challenges	The effects of the activities are an increase of motor skills, volitional qualities, endeavour and readiness to face the challenges given in the process to fulfill the joint group task.

Ref. Number	Full Reference	Title in English	Meth. Code*	Country	Participants			Study Description	Type of Sport	Core Outcomes	Key Findings
					Number	Age	Population				
**115**	Krein, K. (2008). Sport, nature and worldmaking. *Sport, Ethics and Philosophy,* 2(3), pp.285-301	Sport, nature and worldmaking	I	US	n/a	n/a	n/a	Philosophical statement about additional effects of outdoor sports versus traditional sports.	nature sports in general e.g. climbing and surfing	1) enhanced understanding of ourselves, human beings and our relationship to the environment<br>2) worldmaking	The potential of outdoor sport is seen in the engagement of humans in nature and the development of an enhanced understanding of one's self, human beings and our relationship to the environment. Through their action participants create alternative value systems that can contribute to the development of a better world.
**116**	Dettweiler, U., Kugelmann, C., & Streifinger, M. (2011). Expeditionary Learning: Unterwegs auf neuen pädagogisch-didaktischen Pfaden vom Meer bis in die Alpen. In H. Lange, G. Duttler, T. Leffler, A. Siebe & M. Zimlich (Hrsg.), *Bewegungsbezogene Bildungskonzeptionen: zur Trias Konzeption, Implementation und Evaluation* (pp.129-143). Baltmannsweiler: Schneider-Verl. Hohengehren.	Expeditionary Learning: Along the way on new educational pathways from the sea to the Alps.	I	DE	n/i	10 to 14 years	pupils	research hiking tour (2 days, high alpine) and sailing	mountain hiking, sailing	1) academic learning improvements<br>2) social and personal development	The authors propose that learning in the expedition setting promotes personal and social growth as well as academic improvement
**117**	Raichlen, D. A., Bharadwaj, P.K., Fitzhugh, M.C., Haws, K.A., Torre, G.-A., Trouard, T.P. & Alexander, G.E. (2016). Differences in Resting State Functional Connectivity between Young Adult Endurance Athletes and Healthy Controls. *Frontiers in Human Neuroscienc* e, 10(610), pp.1-14.	Differences in Resting State Functional Connectivity between Young Adult Endurance Athletes and Healthy Controls.	C	US	22	18–25 years	male adults	Comparative study on changes in brain structure, function, and connectivity in a sample of endurance runners and a control of non-athletes	endurance cross-country running – focus on intense aerobic exercise	1) changes in brain structure, function, and connectivity<br>2) protective effects of physical activity according to successful ageing	The runners showed greater function connectivity within several areas of the brain, including the frontal cortex. This area is dedicated to cognitive functions like planning, decision-making and the ability to switch attention between tasks. The authors conclude that high intensity aerobic activity that requires sustained, repetitive locomotor and navigational skills may stress cognitive domains in ways that lead to a higher functional brain connectivity. In turn this can lead to a better understanding of the beneficial role of exercise for brain and cognitive function over the lifespan.
**118**	Rogerson, M., Gladwell, V.F., Gallagher, D.J. & Barton, J.L. (2016). Influences of Green Outdoors versus Indoors Environmental Settings on Psychological and Social Outcomes of Controlled Exercise. *International Journal of Environmental Research and Public Health,* 13(4), e363.	Influences of Green Outdoors versus Indoors Environmental Settings on Psychological and Social Outcomes of Controlled Exercise.	B	UK	24	18 to 73 years	adults	Study on psychological and social outcomes of green exercise. The aim is to analyse standardised and yet comparable indoor and outdoor exercise with controlled mode and intensity. Therefore, pairs of participants were randomly assigned to the treatment order and fulfilled two conditions of 15 min cycling on an ergometer placed in an outdoor green environment and inside a laboratory.	cycling on an ergometer placed in indoor and outdoor settings	1) increase of directed attention<br>2) increase of social interaction<br>3) positive influence on intention for future exercise	The study showed that time spent in social interaction was significantly higher in the outdoor setting compared to the indoor condition. Directed attention improved significantly in the outdoor and decreased in the indoor setting. The participants' self-reports showed a strong intention for future exercise in the outdoor condition, but not in the indoor context. Social interaction time can also be seen as a predictor of the intention for future exercise.
**119**	Furman, N. & Sibthorp, J. (2014). The Development of Prosocial Behavior in Adolescents: A Mixed Methods Study from NOLS. *Journal of Experiential Education* , 37(2), pp.160-175.	The Development of Prosocial Behavior in Adolescents: A Mixed Methods Study from NOLS.	C	US	treatment group 57 + comparison group of 60	adolescents	students	Evaluation of a 14-day backpacking course (National Outdoor Leadership School) on prosocial behavior in adolescents	outdoor education programme with backpacking	1) increase of prosocial behavior<br>2) increase of interpersonal skills	The authors suggest that the outdoor intervention increased proximal learning (that which is done with assistance) of prosocial behaviour more than courses featuring a traditional curriculum. This prosocial behaviour was seen as a result of the learning (expedition) environment as working with or understanding others is essential. Post course it can be applied to multiple contexts.
**120**	Sutherland, S. & Stroot, S. (2010). The Impact of Participation in an Inclusive Adventure Education Trip on Group Dynamics. *Journal of Leisure Research,* 42(1), pp.153-176.	The Impact of Participation in an Inclusive Adventure Education Trip on Group Dynamics.	F	US	7	10 to 14 years	youths	Case study of a 3-day inlusive rock climbing trip including one male participant diagnosed with High Functioning Autism	rock climbing	1) inclusion of people with high functioning autism<br>2) group cohesion	Over a 3-day inclusive rock climbing trip the participants bonded as a group. However the authors explain that an explicit teambuilding session conducted by the trip leaders also functioned as a catalyst for the change in group dynamics.

Ref. Number	Full Reference	Title in English	Meth. Code*	Country	Participants			Study Description	Type of Sport	Core Outcomes	Key Findings
					Number	Age	Population				
**121**	Johnson, J. & Chin, J. W. (2016). Seeking new glory (d)hase: A qualitative examinationof adventure based, team orientation rituals as an alternative to traditional sport hazing for athletes and coaches. *International Journal of Sports Science and Coaching* , 11(3), pp.327-341.	Seeking new glory (d)haze: A qualitative examination of adventure-based, team orientation rituals as an alternative to traditional sport hazing for athletes and coaches.	F	US	24	Athletes (19 - 26) coaches (34 - 53)	16 Varsity athletes, 6 coaches	This qualitative study introduced adventure-based alternative orientations to men's and women's collegiate varsity athletic teams. Semi structured interviews were conducted during a weekend intervention with athletes and coaches. Another round of interviews were conducted 6 months after the initial interview.	low rope activities, rock climbing, canoeing	1) sense of cohesion<br>2) communication, shared identity and deeper interpersonal relationships<br>3) alternative to traditional sport hazing for athletes and coaches	Themes that emerged focussed on the transformative effects of the alternative orientations, facilitating a greater sense of cohesion, diminishing team hierarchies, and shifting power relations, effectively democratizing the event and humanizing first year athletes. Participants also felt that alternative orientations played a role in fostering deeper interpersonal relationships rooted in communication and shared identity which they described as facilitating team success.
**122**	Breunig, M., O'Connell, T.S., Todd, S, Anderson, L. & Young, A. (2010). The Impact of Outdoor Pursuits on College Students' Perceived Sense of Community. *Journal of Leisure Research* , 42(4), pp.551–572.	The Impact of Outdoor Pursuits on College Students' Perceived Sense of Community.	D	US	98	19 to 52 years	college students	Effects of an outdoor adventure intervention on the perceived sense of community in college students including a 7 days camp-like residential outdoor education setting and a 6 days wilderness canoe trip.	outdoor adventure education including canoeing	1) increase in sense of community	Findings from this study show that participation in an outdoor education and sports programme result in an increased sense of community in college students. The intervention led to significant changes in the sense of community, reciprocal responsibility, harmony and overall cohesion.
**123**	Wells, N.M. & Lekies, K.S. (2006). Nature and the Life Course: Pathways from Childhood Nature Experiences to Adult Environmentalism. *Children, Youth and Environments,* 16(1), pp.1-24.	Nature and the Life Course: Pathways from Childhood Nature Experiences to Adult Environmentalism.	D	US	2004	18-90 years	adults living in urban areas	Retrospective survey on the influence of childhood nature experiences on environmental attitudes and behavior of adults from a life course perspective	"wild nature" including activities like walking, playing or hiking in natural areas, camping or fishing	1) environmental awareness, pro-environmental attitudes and behavior	The results of the study suggest that experiences of "wild nature" before the age of 11 have a significant, positive association with adult environmental attitudes and behavior.
**124**	Thapa, B., Graefe, A.R. & Meyer, L.A. (2006). Specialization and Marine Based Environmental Behaviors Among Scuba Divers. *Journal of Leisure Research,* 38(4), pp.601-615.	Specialization and Marine Based Environmental Behaviors Among Scuba Divers.	E	US	370	ca. 40 % < 35 years; ca. 34 % > 46 years	recreational scuba diver; 76 % male	Cross-sectional survey on the environmental behavior of recreational scuba divers, no intervention	scuba diving	1) environmentally responsible behavior	Scuba divers reported environmentally responsible behaviour in general. However, the results showed a positive association between the level of specialisation in diving and increased marine based environmentally responsible behaviours.
**125**	North, C. & Harasymchuk, B. (2012). Climbing as if you care: rock climbing at Kura Tawhiti/Castle Hill as a place-based approach to sustainability. *Sports Technology* 5(3/4), pp.132-142.	Climbing as if you care: rock climbing at Kura Tawhiti/Castle Hill as a place-based approach to sustainability.	F	NZ	n/a	n/a	n/a	Theoretical statement on how rock climbing can be used as a means of place-based environmental education	rock climbing	1) placed-based education approach to promote sustainability	Climbing is used as an illustration of how place-based education can promote sustainability. The authors discuss the challenges and opportunities to connect people to places by practicing an outdoor sport and how this can influence environmental behaviours.
**126**	Baena Extremera, A. & Granero Gallegos, A. (2013). Estudio cuasi-experimental de un programa de supervivencia en el medio natural. *Revista Internacional de Medicina y Ciencias de la Actividad Física y del Deporte,* 13(51), pp.551-567.	Quasi-Experimental Study of a program for survival in the natural environment.	C	ES	248 (75 experim ental group / 163 control group)	Univerit y students	University students	The intervention consists of 2 lessons of 4h on survival course in low mountain. Observation of the attitudinal changes in ecological and environmental concerns in environmental awareness and ecocentrism.	survival course in low mountain	1) attitudinal changes in ecological and environmental concerns (short- and long-term)<br>2) increased environmental awareness and ecocentrism (short- and long-term)	Attitudinal changes were observed in ecological and environmental concerns, in environmental awareness and ecocentrism directly after the intervention which was a survival coyurse in the low mountains. This remained through to follow-up measurements four months after the course.
**127**	Inglés, E. & Puig, N. (2016). Gestión de la práctica deportiva en el medio natural. Efectos de la gobernanza en red colaborativa sobre el desarrollo sostenible. *Apunts: Educacion Fisica y Deportes,* 124, pp.89-99.	Sports Management in the Natural Environment: Effects of Collaborative Network Governance on Sustainable Development.	F	ES	35	diverse	people involved in the management of conflict situations in natural protected areas	Analysis of the management of conflicts caused by the practice of outdoor sports in natural protected areas.	trail running, hang-gliding, scuba diving, underwater fishing, kitesurfing, nautic skiing, climbing, hunting	1) increased environmental awareness and ecological concerns	The paper shows the results of the analysis of existing governance in the decision-making processes of eight conflict situations generated by sport in four protected areas in Catalonia. The cohesion in the decision making generates more sustainable impacts on the territory. Outdoor sports can generate changes in environmental awareness and ecological concern on participants and managers of natural protected areas.

Ref. Number	Full Reference	Title in English	Meth. Code*	Country	Participants			Study Description	Type of Sport	Core Outcomes	Key Findings
					Number	Age	Population				
**128**	Rosa, P., Carvalhinho, L. (2012). A educação ambiental e o desporto na natureza: Uma reflexão crítica sobre os novos paradigmas da educação ambiental e o potencial do desporto como metodologia de ensino. *Revista de Educação Física da UFRGS, Movimento,* 18(3), pp.259-280.	Environmental education and nature-based sport: A critical reflection on the new paradigms of environmental education and the potential of sport as a teaching methodology.	H	PT	n/a	n/a	n/a	Literature review that critically reflects on nature sports and its potential as a teaching methodology.	surfing, hiking, nature-based sports in general	1) increased environmental awareness<br>2) tool for environmental education	Based on the critical reflection of existing literature the authors come to the conclusion, that nature-based sports can be used as a tool for environmental education. As nature-based sport can go beyond the mere transmission of knowledge it has a potential to enhance pro-environmental behaviour in the context of situated and experiential learning.
**129**	Luthe, T., Häusler, R., & Roth, R. (2007). Die Durchführung alternativer Schneesportausfahrten und deren Nutzung zur Bildung für eine nachhaltige Entwicklung (BfnE). In *Sportunterricht,* 56 (12), pp.366-370.	Alternative snow sport camps and their potential for education for sustainable development	F	DE	n/i	young generation	young generation	study reflects on a concept for educationally meaningful snow sport activities	snow sports	1) motivating and attractive method for teaching sustainability	Snow sports can be used as attractive and motivational method to interest young people in the topic of sustainability and to teach and understand this complex construct in an appealing way with a lasting effect
**130**	Limmer, M., & Roth, R. (2016). Effects of a 5-day outdoor sports intervention on environmental attitudes in children. in A. Baca (Hrsg.): *Book of Abstracts - 21. Annual Congress of the European College of Sport Science : Crossing borders through sport science* (p.520). European College of Sport Science.	Effects of a 5-day outdoor sports intervention on environmental attitudes in children.	D	DE	288	11-14 years	students	study on the impact of a 5-day extra-curricular outdoor sport interventions on environmental attitudes in children	various outdoor sports	1) environmental awareness and attitudes	The 5-day extra-curricular outdoor sports programs showed a positive short-term effect on the students' environmental attitudes; Interventions seemed most effective for children from urban areas and for secondary general school students who had a lower level of academic prowess.
**131**	Thorpe, H. (2016). Action sports for youth development: critical insights for the SDP community. *International Journal of Sport Policy* . 8(1), pp.91-116.	Action sports for youth development: critical insights for the SDP community.	F	NZ	n/a	n/a	n/a	Descriptions of the author's experiences and case studies/projects, international examples and qualitative research including interviews and media analysis	skateboarding, surfing, snowboarding, parkour	1) contribution to sport for development and peace	Based on qualitative research and analysis of various case studies and projects, the author showcases the potential of action sports for making a valuable contribution for youth development and engagement as well as to the sport for development and peace movement.
**132**	Coulom, J-C. & Bessy, O. (2014). Du Stade d'Eaux Vives Pau-Pyrénées au Parc Naturel Urbain: Entre récréativité innovante et innovation territoriale. *Loisir et Société / Society and Leisure* , 37(1), pp.79-100.	White water stadium of Pau (south west of France - Pyrénées) and Urban natural Park: In between new trends of outdoor leisure and territorial innovation.	F	FR	n/a	n/a	n/a	Case study in the field of geographical planning. It showcases how outdoor sports can be achored in an urban area and what different roles they can play in the construction of the latter.	white water rafting	1) improved urban recreation<br>2) revitalization and recomposition of urban territories	The study shows the efforts undertaken by the city to develop an attractive and innovative urban recreation venue through the use of outdoor sports. It showcases how a white water rafting stadium can be developed to revitalise and re-shape urban territories through the provision of an attractive natural space, especially for people living on the outskirts of town.
**133**	Baena Extremera, A. & Granero Gallegos, A. (2008). Las actividades físicas en la naturaleza en el currículum actual: contribución a la educación para la ciudadanía y los derechos humanos. *Retos: nuevas tendencias en educación física, deporte y recreación,* 14, pp.48-53.	Outodoor activities in the present curriculum: contribution to the education for citizenship and human rights.	F	ES	n/a	n/a	students.	Outdoor activities are discussed as an educational method for the learning of citizenship and human rights. It consists of a proposal of ideas and actions to increase citizenship and human rights conscience in students.	oudoor sports in general	1) increased citizenship and human rights<br>2) educational tool for the learning of citizenship and human rights	A set of proposals, ideas and actions were developed to apply outdoor activities to enhance the learning of citizenship and human rights.

Ref. Number	Full Reference	Title in English	Meth. Code*	Country	Participants			Study Description	Type of Sport	Core Outcomes	Key Findings
					Number	Age	Population				
134	Barnes, M., Forrester, S. & Walsh, M. (2008). Leisure as community catalyst: Re-visiting a community twenty years after the development of a municipal artificial white water river. *Proceedings of the World Leisure Congress,* 10th , Quebec City. Oral presentation 94, p.26.	Leisure as community catalyst: Re-visiting a community twenty years after the development of a municipal artificial white water river.	F	CA	n/a	n/a	n/a	qualitative study on the effects of a municipal artificial white water river development on a local community twenty years after it's inception	white water canoeing, kayaking, rafting and tubing	1) re-development of a decaying urban downtown 2) source of community identity and pride	The qualitative analysis shows how a community has been successful in using an artificial white water river as a catalyst for local development over a twenty year period. As a conclusion of the social, cultural, economic, and environmental benefits the authors highlight 4 key themes: 1. the natural extension of a well-known park system; 2. The re-development of a decaying urban downtown; 3. A water feature for recreational, instructional and competitive canoeing, kayaking, rafting and tubing; and 4. A source of community identity and pride.
135	Goodwin, D., Peco, J. & Ginther, N. (2009). Hiking Excursions for Persons with Disabilities: Experiences of Interdependence. *Therapeutic Recreation Journal,* 43(1), pp.43-55.	Hiking Excursions for Persons with Disabilities: Experiences of Interdependence.	F	CA	4	aged 27, 28, 36 and 54.	participants had complete spinal cord injuries (two quadriplegic, two paraplegic)	Phenomenological case study to understand the experience of participating in a group-assisted outdoor hiking excursion from the perspective of hikers with disabilities. An 8 week hiking program for people with disabilities was provided. Pre and post semi-structured interviews were conducted and participants took photographs and field notes.	hiking	1) inclusion of disabled	Four themes were revealed in the thematic analysis: 'Off'road rush' 'not a wheelbarrow' 'loss of control' and 'interdependence'. The exhilarating hiking experiences were accented by the transfer of individual control and independence of the hikers to the larger team.
136	McNamee, J. & Timken, G. (2017). Outdoor Pursuits in Physical Education. Lessons from the Trenches. *Journal of Physical Education, Recreation & Dance* , 88(3), pp.8-15.	Outdoor Pursuits in Physical Education. Lessons from the Trenches	I	US	n/a	n/a	students	Qualitative paper on insights from 10 teachers who offer outdoor activities	outdoor activities in general including land (e.g., mountain biking, rock climbing, snow shoeing, ...) and water activities	1) realistic connections to lifetime activity habits 2) increased chances for children for lifetime of physical activity participation	According to the teachers' valuation and expert opinion outdoor activities offer better chances than traditional team sports for children to find and pursue life-long physical activity as they have more realistic connections to lifetime activity habits in the adult population.
137	Rosa, P., Teixeira de Almeida, M. & Carvalhinho, L. (2013). Gestão do desporto de natureza em áreas protegidas: estudo de caso no PNSCA. *Revista Intercontinental de Gestão Desportiva,* 3(1), pp.1-15.	The management of nature-based sports in protected areas: case study in PNSAC.	E	PT	82	adults	All the participants were practitioners of nature sports and residents in Rio Maior area	Cross-sectional survey on the perception of practitioners of outdoor sports and residents regarding: 1) potential of the area for outdoor sports practice. 2) characteristics of the athlete and their practice in the designated area. 3) social and environmental problems related with the practice of outdoor sports 4) opinions regarding the management of outdoor sports practice in the area.	horse riding, ballooning, mountain bike, canoeing, climbing, orienteering, caving, paragliding, hiking	1) no social conflicts between different nature sports activities 2) limited damage of natural environment	Generally there were no critical social conflict cases identified regarding the practice of different nature sports in the same area. Regarding the environmental impact of nature based sports, no participants evaluated any significant physical degradation of the area, but over 44% highlighted some degradation while 42% saw no alteration. 15% felt that the environment had improved.
138	Mieras, M. E., Heesch, M. W. S. & Slivka, D. (2014). Physiological and psychological responses to outdoor vs. laboratory cycling. *Journal of Strengt and Conditioning Research,* 28(8), pp.2324-2329.	Physiological and psychological responses to outdoor vs. laboratory cycling.	C	US	12	mean age of 37	recreationally trained male road cyclists	A study to assess the physiological and psychological responses to laboratory vs. outdoor cycling. Participants had two experimental trials with no fewer than 2 days and no longer than 2 weeks between the sessions. First was in a laboratory setting, riding their own bikes on an electronically braked cycle trainer ergometer. After a 10min warm up at 150 W they rode at a workload of 95 W, the workload increased by 35W every 3 minutes until volitional fatigue.	indoor (laboratory) / outdoor road cycling	1) outdoor settings allow higher intensity of exercise	Power output and HR were higher in the outdoor trial than lab. Core temperature remained similar whereas skin temperature was cooler in the outdoor trial. No significant difference in bodyweight, RPE (rate of percieved exertion) or USG (urine specific gravity) was found. These results indicate that outdoor cycling allows cyclists to exercise at a higher intensity that in a laboratory setting despite similar environmental conditions and percieved exertion.

Meth. Code: A = Systematic reviews/ Meta-analyses; B = Randomised Controlled Trials (RCTs); C = Case-control studies; D = Longitudinal studies; E = Cross-sectional surveys; F = Case reports/study/ programs/ qualitative evaluations; G = Economic evaluations;
H = Narrative, non-systematic review; I = Policy statement, theoretical paper
