# Supplementary material for: Benefits of Outdoor Sports for Society. A Systematic Literature Review and Reflections on Evidence
Source: Int J Environ Res Public Health. 2019 Mar 15;16(6):937. doi: 10.3390/ijerph16060937 (PMC6466442; doi:10.3390/ijerph16060937)
Supplement: Supplementary file 1 [file ijerph-16-00937-s001.zip › IJERPH_S3.pdf]

S3: Origin of selected studies (n=133)

Country	AT	AU	BE	BG	CA	CH	CN	CZ	DE	DK	ES	FI	FR	GR	HR	IE	IT	NL	NZ	PT	RO	SI	TR	TW	UK	US
total number	2	8	1	3	10	2	2	1	10	1	7	1	1	3	1	1	5	1	3	5	1	1	1	2	25	35
